# A Temporal Activity of CA1 Neurons Underlying Short-Term Memory for Social Recognition Altered in PTEN Mouse Models of Autism Spectrum Disorder

**DOI:** 10.3389/fncel.2021.699315

**Published:** 2021-07-15

**Authors:** An-Ping Chai, Xue-Feng Chen, Xiao-Shan Xu, Na Zhang, Meng Li, Jin-Nan Li, Lei Zhang, Dai Zhang, Xia Zhang, Rong-Rong Mao, Yu-Qiang Ding, Lin Xu, Qi-Xin Zhou

**Affiliations:** ^1^Key Laboratory of Animal Models and Human Disease Mechanisms, and Laboratory of Learning and Memory, and KIZ-SU Joint Laboratory of Animal Model and Drug Development, Kunming Institute of Zoology, The Chinese Academy of Sciences, Kunming, China; ^2^The Brain Cognition and Brain Disease Institute, Shenzhen Institute of Advanced Technology, Chinese Academy of Sciences, Shenzhen, China; ^3^Shenzhen-Hong Kong Institute of Brain Science-Shenzhen Fundamental Research Institutions, Shenzhen, China; ^4^School of Life Sciences, Yunnan University, Kunming, China; ^5^School of Life Sciences, Anhui University, Hefei, China; ^6^Department of Anatomy and Neurobiology, Tongji University School of Medicine, Shanghai, China; ^7^Institute of Mental Health, The Sixth Hospital of Peking University, Beijing, China; ^8^Department of Cellular and Molecular Medicine, Institute of Mental Health Research at the Royal, University of Ottawa, Ottawa, ON, Canada; ^9^Department of Psychiatry, Institute of Mental Health Research at the Royal, University of Ottawa, Ottawa, ON, Canada; ^10^CAS Center for Excellence in Brain Science and Intelligence Technology, Shanghai, China

**Keywords:** short-term (working) memory, social recognition, phosphatase and tensin homolog, hippocampal CA1, optogenetic tool, autism (ASD)

## Abstract

Memory-guided social recognition identifies someone from previous encounters or experiences, but the mechanisms of social memory remain unclear. Here, we find that a short-term memory from experiencing a stranger mouse lasting under 30 min interval is essential for subsequent social recognition in mice, but that interval prolonged to hours by replacing the stranger mouse with a familiar littermate. Optogenetic silencing of dorsal CA1 neuronal activity during trials or inter-trial intervals disrupted short-term memory-guided social recognition, without affecting the ability of being sociable or long-term memory-guided social recognition. Postnatal knockdown or knockout of autism spectrum disorder (ASD)-associated phosphatase and tensin homolog (*PTEN*) gene in dorsal hippocampal CA1 similarly impaired neuronal firing rate *in vitro* and altered firing pattern during social recognition. These PTEN mice showed deficits in social recognition with stranger mouse rather than littermate and exhibited impairment in T-maze spontaneous alternation task for testing short-term spatial memory. Thus, we suggest that a temporal activity of dorsal CA1 neurons may underlie formation of short-term memory to be critical for organizing subsequent social recognition but that is possibly disrupted in ASD.

## Introduction

A vast amount of short-term memories (STM) are formed during social interaction, but most of them hold information only temporally and decay instantly without transferred into long-term memories (LTM). In contrast, working memory (WM) as a particular type of STM is suggested to be a cognitive process not only with capacity limitation in holding and processing necessary information temporally, but also able to manipulate and/or supervise LTM (Baddeley, 2012). STM thus differs from WM but both share a common feature by holding information temporally (Diamond, 2013; Rose et al., 2016).

Extensive studies have harvested fruitful understanding of neuronal mechanisms on WM and LTM. The prefrontal cortex, the cingulate cortex, and the hippocampus, etc. are involved in WM (Wager and Smith, 2003; Olson et al., 2006). WM theories have suggested persistent neuronal activity as its major mechanism (Fuster and Alexander, 1971; Otto and Eichenbaum, 1992; Goldman-Rakic, 1995; Liu et al., 2014). In marked contrast, synaptic plasticity such as long-term potentiation (LTP) or long-term depression (LTD) in these brain regions has been believed as at least one of the key mechanisms underlying LTM (Bliss and Collingridge, 1993; Malenka and Nicoll, 1999; Martin et al., 2000). Nevertheless, the underlying molecular and cellular mechanism for STM remains largely unclear.

STM, WM, and LTM are all critical for our daily social behaviors because, without normal function of them, one can obviously predict that social behaviors such as social recognition, communication, and interaction with others will be problematic (Smith and DeCoster, 2000; Spreng, 2013). It has been reported that short-term face memory and WM are impaired in autism spectrum disorder (ASD) (Poirier et al., 2011; Weigelt et al., 2012; Barendse et al., 2013). ASD is a highly inheritable neurodevelopmental disorder, marked by deficits of social behaviors (Williams et al., 2005; Crane and Goddard, 2008; Levy et al., 2009). Among the risk genes associated with ASD, mutations in *PTEN* (phosphatase and tensin homolog deleted on chromosome 10) have been defined, which count for about 5–10 % of ASD children with macrocephaly (Butler et al., 2005; Buxbaum et al., 2007), and for about 80 % of classic Cowden syndrome (Goffin et al., 2001; Eng, 2003). ASD children with *PTEN* mutations exhibit deficits of social behaviors and STM while working on a task (Eng, 2003; Frazier et al., 2015). It is important to note that these deficits have been replicated in mice with PTEN conditional knockout (CKO) in the cerebral cortex and the hippocampus since early postnatal stage (Kwon et al., 2006; Zhou et al., 2009). Although social behavior impairments may attribute to neural morphological abnormalities due to the up-regulated phosphatidylinositol 3-kinase signaling (Kwon et al., 2001; Fraser et al., 2008; Zhou et al., 2009; Jia et al., 2010) or altered synaptic plasticity and/or neuronal activity (Kwon et al., 2006; Garcia-Junco-Clemente et al., 2013; Takeuchi et al., 2013), the mechanisms underlying social recognition deficit remain to be elucidated.

Lesions and pharmacological studies revealed that dorsal hippocampal CA1 was essential for duration-dependent STM (Friedman and Goldman-Rakic, 1988; Otto and Eichenbaum, 1992; Lee and Kesner, 2003; Izaki et al., 2008; Zhang et al., 2013). The dorsal hippocampal CA1 recruitment in memory-guided social recognition is still controversial (Lai et al., 2005; von Heimendahl et al., 2012; Pena et al., 2014). Notably, recent studies found that social locations were represented in activity of dorsal hippocampal CA1 neurons (Danjo et al., 2018; Omer et al., 2018). However, optogenetic and chemogenetic studies also shed light on crucial role of ventral hippocampal CA1 and dorsal hippocampal CA2 in LTM-guided social recognition (Hitti and Siegelbaum, 2014; Okuyama et al., 2016). Hence, dorsal hippocampus CA1 region may have a critical role of regulating STM-guided social recognition.

Here we addressed what the underlying mechanism is for memory-guided social recognition and its deficit induced by PTEN-loss. In this study, we found that the interval from experiencing a stranger mouse to subsequent social recognition test was limited by a short time period of less than 30 min. With this behavioral model, optogenetic silencing of dorsal CA1 neuronal activity during the experiencing or the interval similarly disrupted subsequent social recognition, suggesting that a temporal activity of dorsal CA1 neurons is critical for STM processing in organization of social recognition. On the other hand, our previous study revealed that knockout (KO) of *P-REX1*, an ASD-associated gene found in Chinese Han children, lead to compromised social recognition and dorsal CA1 synaptic plasticity, but normal dorsal CA1 neuronal firing *in vitro* (Li et al., 2015). Using similar method, however, we here found that dorsal CA1 neurons from PTEN CA1 knockdown (KD) mice exhibited reduced firing rate *in vitro*, or disrupted firing pattern of dorsal CA1 neurons from CaMKII-Cre × *Pten*^*loxP/loxP*^ mice during social recognition test without affecting dorsal CA1 synaptic plasticity. Notably, postnatal PTEN deletion in these mice lead to similar deficits in STM-guided social recognition or T-maze spontaneous alternation task for testing spatial STM, but sociability, social recognition with familiar littermate and a GO/NO GO task were unaffected. Thus, we define that a temporal activity of dorsal CA1 neurons may underlie STM for organizing social recognition but that is possibly disrupted in PTEN mouse models of ASD.

## Materials and Methods

### Animals

Male C57BL/6 mice (from Vital River Laboratory Animal Technology Co. Ltd., Beijing, China), aged 6–9 weeks, were used. The CaMKII-Cre mice (Mayford et al., 1996; Tsien et al., 1996b) from Prof. Yuqiang Ding (Tongji University) were crossed with *Pten*^*loxP/loxP*^ mice (Lesche et al., 2002) from Prof. Xudong Zhao (Kunming Institute of Zoology, CAS) for generating *Pten* conditional knockout (CKO) mice. Age-matched unfamiliar conspecific male stranger mouse and subject mouse are raised in separate cage. Familiar littermate mouse and subject mouse are raised in same cage for at least 3 weeks before test. Each cage contains 3–5 mice. Experimental protocols were approved by the Institutional Animal Care and Use Committee of Kunming Institute of Zoology, the Chinese Academy of Sciences.

### Lentivirus Construction and Injection

The lentivirus (LV) construction (LV-shRNA-PTEN-EGFP) was from Shanghai GeneChem. Co., Ltd. The *Pten* shRNA targeted the sequence (AGAGATCGTTAGCAGAAAC) was cloned from a pSilencer3.1-H1 vector from Prof. Xia Zhang (Jia et al., 2010). The negative control (LV-shRNA-NC-EGFP), a scrambled sequence (TTCTCCGAACGTGTCACGT) was also from the same company. These sequences were ligated into GV118 (U6-MCS-Ubi-EGFP) lentiviral vector using the HpaI/XhoI sites. The viruses (>10^9^ TU/ml) were used in the present study.

For dorsal CA1 viral injections, C57BL/6 mice aged 3–4 weeks were anesthetized with pentobarbital (70 mg/kg, i.p.) and placed in a stereotaxic apparatus (RWD Life Science Co., Ltd., Shenzhen, Guangdong, China). Using two 5 μL micro syringes (Hamilton, Reno, NV, United States) with two 30 gauge needles (RWD Life Science Co., Ltd., Shenzhen, Guangdong, China), 1.5 μL of the viruses was delivered at 0.2 μL/min by a micro-syringe pump (RWD Life Science Co., Ltd., Shenzhen, Guangdong, China) at two sites in each of the bilateral CA1 regions, using the stereotaxic coordinates: 1.5 mm (anterior-posterior) from bregma, 1 mm (medio-lateral), ± 1.5 mm (dorso-ventral) for site 1, and 2.5 mm (anterior-posterior) from bregma, ± 2 mm (medio-lateral), 1.5 mm (dorso-ventral) for site 2. The syringe was left in place for 5 min after each injection and withdrawn slowly. The exposed skin was closed by surgical sutures and returned to home cage for recovery. All the experiments were conducted after at least 3 weeks of recovery. All the mice were sacrificed after experiments to confirm the injection sites and the viral trans-infection effects by checking EGFP under a fluorescence microscope (600-FN, Nikon, Japan).

### Immunohistochemistry

X-gal staining of CaMKII-Cre;Rosa26-LacZ mouse brain sections was performed as previously reported (Hu et al., 2012). Briefly, mice were perfused with 0.01M PBS, followed by 4% paraformaldehyde (PFA) in 0.1 M phosphate buffer (PB, pH = 7.4). Brains were dissected out and fixed in 4% PFA for 1 h and then moved to 30% sucrose in PBS for cryoprotection. Brain sections of 30 μm were obtained using a cryostat (CM1900, Leica) and incubated in X-gal staining solution [0.1 M PB (pH = 7.4) containing 1 mg/ml X-gal, 2 mM MgCl_2_, 5 mM K_3_Fe(CN)_6_, and 5 mM K_4_Fe(CN)_6_)] for 10–16 h at 37°C. Sections were then mounted onto gelatin-coated glass slides and counterstained with neutral red. Images were taken with a microscope (Eclipse 80i, Nikon).

### Immunofluorescence Staining

Mice were perfused with 0.01M PBS, followed by 4% paraformaldehyde (PFA) in 0.1 M phosphate buffer (PB, pH = 7.4). Brains were dissected out and fixed in 4% PFA for 12 h and then moved to 30% sucrose in PBS for 48 h. After sinking to the bottom of vial, the whole brain was cut into brain block containing hippocampus and embedded in 4% agarose. Brain sections of 40 μm were obtained by using a vibrating microtome (VT1000s, Leica). The slice sections were incubated with rabbit anti-PTEN (1:200; Abcam; ab32199) and mouse anti-NeuN (1:200; Millipore Bioscience Research Reagents; MAB377). Primary antibodies were detected using secondary antibodies conjugated with Cy3 (1:1000; Jackson ImmunoResearch) or DyLight405 (1:1000; Jackson ImmunoResearch). Images were taken with a microscope (FV1000, Olympus).

### Western Blot

PTEN expression in CA1 regions was tested 3-week after virus injection, or in *PTEN* CKO mice at 8 weeks old. The samples were collected from bilateral dorsal hippocampi (WT and *Pten* CKO) or naïve mice (three mice in one sample) or bilateral dorsal CA1 affected by the virus (shRNA-PTEN and shRNA-NC) in 12 mice under fluorescence microscope (three mice for one sample) and lysed by using homogenizer in ice-cold RIPA buffer (50mM Tris-HCl (pH7.4), 1% NP-40, 0.5% sodium deoxycholate and 0.1% SDS and protease and phosphatase inhibitors). The homogenates were centrifuged at 12,000 rpm for 15 min at 4°C. Discard the precipitate and leave the supernatant. Protein concentration was determined by using the bicinchoninic acid protein assay kit (BCA kit, Tiangen Biotech Co. LTD, Beijing, China). Then the samples were aliquoted and stored at −80°C. PTEN expression level in total protein (15 μg/sample) was assessed by following a previously described protocol (Jing et al., 2015). The primary antibodies used for detection were rabbit anti-PTEN (1:2000, no. ab32199, Abcam, Cambridge, MA, United States), rabbit anti-GAPDH (1:10000, KangChen Bio-tech Inc., Shanghai, China). Blots were probed with secondary antibody goat anti-rabbit HRP (1:15000, KangChen Bio-tech Inc., Shanghai, China). The protein expression level was quantified using X-ray densitometry and scanned by EPSON ME office 700FW scanner at a 1200 dpi resolution and analyzed using ImageJ software (National Institutes of Health, Bethesda, MD, United States).

### Optogenetic Study

The viruses (pAAV-CaMKIIα-NpHR3.0-EYFP and/or pAAV-CaMKIIα-EYFP) were obtained from Obio Technology CO., Ltd (Shanghai, China). The virus of more than 10^12^ TU/ml was used for CA1 injection. The procedure of AAV injection was the same as the injection of lentivirus using the coordinates: 2 mm (anterior-posterior) from bregma, 1.5 mm (medio-lateral), 1.5 mm (dorso-ventral). After the injection, two implantable fiber-optic cannulas made by ceramic ferrule (Φ1.25 mm) with multimode fiber (NA = 0.39, Φ = 200μm, *L* = 5 mm; Thorlabs, Inc. Newton, NJ, United States) were immediately implanted 0.3 mm above the injection sites. Then the optical fibers were fixed to the skull surface by using cyanoacrylate glue. Afterward, the ceramic ferrules were secured by dental cement. All the behavioral and electrophysiological experiments were conducted after 3 weeks recovery. All mice used in social interaction test were sacrificed to confirm the injection sites and virus trans-infection effect by checking EYFP under fluorescence microscope (600-FN, Nikon, Japan).

### Three-Chamber and Three-Trial Social Interaction Test

Social interaction test was conducted as previously described (Nadler et al., 2004; Moy et al., 2007; Li et al., 2015). The box (62 cm L x 40 cm W x 22 cm H) was divided by two equally spaced white Plexiglas panels with retractable rectangular doorway (5 cm W x 22 cm H) allowing free access into each chamber (20 cm L x 40cm W x 22 cm H), for the convenience of applying multi-electrode recording and optogenetics in three-chamber social interaction experiments. Each of the two side chambers contained an empty wire cylindrical Plexiglas cage (15 cm H, 10 cm bottom diameter, and 15 bars spaced 1 cm apart) at the corner of same side to enclose strangers. At the end of each test, the apparatus was cleaned with 75% ethanol and dried with paper towels. Behavioral testing was carried out in a sound-proof, dimly lit (less than 20 lux) animal behavioral test room.

The standard three-chamber social interaction test includes three trials, each for 10-min. The subject mouse was first placed in the middle chamber and allowed to explore all chambers for 10-min habituation. After trial 1, the doorway was closed and the subject mouse was kept in the middle chamber. An age-match unfamiliar conspecific male mouse (stranger 1, S1) was enclosed in one of the wire cages. Then, the doors were raised again and the subject mouse was free to explore the chambers for a 10-min session. After trial 2, the subject mouse was kept in the middle chamber again and a second stranger mouse (stranger 2, S2) was placed in another wire cage. The subject mouse then was left to explore the chambers for another 10 min session in trial 3. All the social processes were videotaped from above and stored by Ethovision XT8 video-tracking system (Noldus Information Technology, Wageningen, Netherlands). The sniff zone was defined as proximity (2 cm) with the cylindrical cage. The time of directing the nose to the stranger within sniff zone and/or touching the cage with the nose was hand scored by a skilled technician who was blind to the genotypes. The positions of stranger 1 were alternated between left and right sides across each of the subject mice. Each stranger was placed in the cages no more than three times each day.

For the delay-dependent social memory test, there was an inter-trial interval (ITI) of 1 min, 5 min, 30 min, or 6 h between trial 2 and trial 3. In within-subject test, each subject mouse was tested once a week but twice at most. The ITI between trial 2 and trial 3 in the rest of three-chamber social interaction tests was 5 min unless mentioned elsewhere. During ITI 1 min, 5 min, and 30 min, subject mouse was kept in the middle chamber. During ITI 6 h, subject mouse was returned to home cage, the selected familiar littermate, the subject mice from the same cage and the strangers were kept separately with food and water access *ad libitum*. For position-dependent social memory test, at the end of trial 2, stranger 2 (S2) was placed into the cage which enclosed stranger 1 in trial 2; stranger 1 (S1) was switch to the cage which was empty in trial 2. For novelty-dependent social memory test, at the end of trial 1, a littermate male mouse (familiar, F) was enclosed in one of the empty cages; after trial 2, a stranger mouse (S) was placed in the wire cage that had been empty in trial 2. For position-independent social memory test, at the end of trial 2, a stranger mouse (S) was placed into the cage which enclosed familiar mouse (F) in trial 2; the familiar mouse (F) was switched to the cage which was empty in trial 2. For behavioral characteristics of stranger 2 in social interaction, a mouse-sized wooden object (Ob), or a familiar mouse (F) was treated as stranger 2 respectively. All the stranger 1, stranger 2 and familiars were termed as stimuli mice.

Three weeks after the injection of pAAV-CaMKIIα-NpHR3.0-EYFP and/or pAAV-CaMKIIα-EYFP, two optical fibers (NA = 0.37, Φ = 200 μm; Fiblaser, Shanghai, China) linked to a communicator was connected to the pre-planted ceramic ferrules. The light pulse given by a yellow light laser (MBL-II, Brain-King Limited Co., Beijing, China) was controlled by a stimulator (GRASS S88X, Astro-Med, Inc, West Warwick, United States).

### T-Maze Spontaneous Alternation Test

A T-maze apparatus (Med Associates Inc., United States) was used for mice. The mouse was first put into the start area of the start arm to habituate for 15 s, then the door was lifted to let the mouse walk freely toward the goal arms. After the mouse chose and entered one goal arm with its four paws and tail, it was limited to the selected goal arm for 15 s by guillotine door. This was the sample run and the direction of the sampled goal arm was recorded. Then the mouse was transferred back to the start area of the start arm with a cylinder cardboard and kept in the start area for 1 min. After that, the door of the starting area in the starting arm was lifted to let the mouse walk freely toward the goal arms. If the mouse chose and entered the unsampled goal arm, indicating correct memory of the sample run. This stage was called the choice run. After the experiment, the mouse was returned to the cage, and the maze was wiped with 75% alcohol and dried with paper towels. Each mouse was tested twice a day for seven consecutive days, once in the morning and once in the afternoon, that is 14 tests in total.

### Object Recognition Test

This test was performed following the protocols previously described (Bevins and Besheer, 2006; Pavlopoulos et al., 2013; Li et al., 2015). For novel object preference task, two identical objects were firstly placed abreast with 9 cm distance from the wall of a customized black Plexiglas box (40 cm L x 30 cm W x 40 cm H). The mouse was placed facing the wall in the box to freely run in the box for a 20 min training session. After training, animal was taken back to home cage. Followed by 5 min delay, one of the identical objects was replaced by a novel object, and the mouse was placed back to the box again and allowed for a 10 min test session. For object location task, after 20 min training on two identical objects, one of the objects were moved to a diagonal corner compared with the other identical subject, and the mouse was tested for a 10 min session. Two pairs of objects were used. Two asparagus-shape transparent soft-plastic bottles filled with black plastic particles (maximal diameter 6 cm and total height 18 cm). The whole experiments were recorded by Ethovision XT8 video-tracking system (Noldus Information Technology, Wageningen, Netherlands). Time of directing the nose to the object within 2 cm distance and/or touching object with the nose were hand scored by human observer with stopwatches. At the end of each test, the box was cleaned with 75% ethanol and dried with paper towels. Behavioral tests were carried out in a sound-proof room with dim light (less than 20 lux).

### Buried Food Test

The test was carried out as previously reported (Yang and Crawley, 2009; Li et al., 2015). One day before the test, food stimuli (3–5 peanuts) were put in the home cage in the morning. Conforming the consumption of food stimuli before dusk, that is, 14 to 18 h before the test. All the food was removed from the home cage except for water. On the next day, each subject mouse was placed in a clean cage (29 cm L x 21 cm W x 16 cm H) containing a 3-cm depth of cleaning bedding and allowed to acclimate to the cage for 5 min. Then, the subject was transferred to another temporary holding cage when the food stimulus was buried nearly 1 cm beneath the surface of bedding, in a random corner of the habituated cage. The subject was re-placed into the testing cage from temporary holding cage and left to find the buried food for at most 15 min. The latency to uncover and start eating the food was recorded. Latency was recorded as 900 s if the mouse did not find the food. All the food and water were replaced and fulfilled upon completion of the test.

### Slice Preparation

For extracellular field recording, hippocampal slices were prepared from mice using procedures described previously (Li et al., 2015). The animal was deeply anesthetized with isoflurane. After decapitation, the brain was taken rapidly and sectioned into 350 μm coronary slices by using a vibratome (Leica VT1000S, Leica Microsystems, Germany) in ice-cold oxygenated (95% O_2_/5% CO_2_) cutting solution containing (in mM): 206 sucrose, 2.5 KCl, 1.25 NaH_2_PO_4_, 26 NaHCO_3_, 10 D-glucose, 2 MgSO_4_7H_2_O, 2 CaCl_2_H_2_O (pH 7.2–7.4, 290–300 mOsm). Slices were then transferred into a holding chamber with 32°C oxygenated (95% O_2_/5% CO_2_) artificial cerebral spinal fluid (ACSF) solution containing (in mM): 120 NaCl, 2.5 KCl, 1.25 NaH_2_PO_4_, 26 NaHCO_3_, 10 D-glucose, 2 MgSO_4_7H_2_O, 2 CaCl_2_H_2_O (pH 7.2–7.4, 290–300 mOsm) for 45 min. Then the slices in the incubation chamber were kept at room temperature (RT) for at least 1 h before recording.

### *In vitro* Patch-Clamp Recording

The field excitatory postsynaptic potentials (fEPSPs) in the dorsal hippocampus CA1 area were recorded as described in our previous study (Dai et al., 2008). All recordings were carried out in the recording chamber which was maintained at RT with standard ACSF and was placed on the stage of the Nikon microscope (600-FN, Nikon, Japan). The stimulating electrode was made by a pair of twisted Teflon-coated 90% platinum/10% iridium wires (0.025 mm diameter, World Precision Instruments). Recording electrode (1 MΩ) was pulled from borosilicate glass capillaries (1.5 mm outer diameter, 0.84 mm inner diameter, World Precision Instruments) with a Brown-Flaming micropipette puller (P-97; Sutter Instruments Company, America) and filled with standard ACSF. The fEPSPs were recorded in the stratum radiatum of the dorsal CA1 area of the hippocampus by stimulation (0.1 ms duration) of the Schaffer collaterals (SCs). The fEPSP in SC-CA1 pathway is primarily involved in excitatory glutamate receptors (e.g., AMPA receptor and NMDA receptor) (Davies and Collingridge, 1989). The GABA-dependent inhibitory transmission was not blocked during fEPSP recording. For input-output recordings, fEPSP slopes were recorded by increasing the stimulation (0.1 ms pulse width) intensity from 0 in 20 μA increments. For paired-pulse ratio (PPR), the stimulation intensity was adjusted to give fEPSP slope of 50% of maximum, a pair of stimuli with different intervals including 50 ms, 100 ms, 150 ms, 200 ms, and 1000ms was given successively for three times. The average value of three successive rounds was used for PPR analysis. The PPR was calculated as the ratio of the slope of the 2nd fEPSP to the slope of the 1st fEPSP. For short-term augmentation (STA) induction, 30 stimuli at intensity of 50% of the maximal fEPSPs slope were given at 20 Hz. For long-term potentiation (LTP) induction, a stable baseline was established with a stimulation strength which was set to elicit a response equivalent to about 40–50% of the maximal fEPSPs slope and maintained for at least 20 min at 0.033 Hz. Only slices that maximal amplitude was more than 1 mV were included in the study. Then LTP was induced by high-frequency stimulation (HFS, 3 trains of 1-sec stimulation at 100 Hz with 20s inter-train intervals) with the same stimulation intensity which was used for baseline recording.

For whole-cell recording, pyramidal neurons in CA1 were identified under infrared-differential interference contrast (IR-DIC) microscope (600-FN; Nikon, Japan). For action potential recording, the recording pipettes (3–6 MΩ) were filled with the internal solution containing (in mM):130 potassium gluconate, 6 NaCl, 20 HEPES, 0.2 EGTA, 1 MgCl_2_, 2 MgATP, 0.3 Na_3_GTP (pH 7.2–7.4 with KOH, 280–290 mOsm). Spikes were induced by current injection from 0 to 350 pA for 1s in 50 pA increments. In order to verify the NpHR function, action potentials evoked in neuron affected by pAAV-CaMKIIα-NpHR3.0-EYFP by injecting currents were abolished by concomitant 8–10 mW yellow light illumination (594 nm) from a yellow light laser (MBL-II, Brain-King Limited Co., Beijing, China). Signals were acquired with Multiclamp 700B and Digidata 1440A (Molecular Devices, Sunnyvale, CA, United States). Data were filtered at 2 kHz and sampled at 10–20 kHz. Series resistance was smaller than 25 MΩ and constantly monitored. Neurons with change in series resistance less than 20% were included in the analysis. Liquid junction potential (ca. 5.8 mV) was corrected during experiments. All the data were recorded by using pCLAMP 10 software (Molecular Devices, Sunnyvale, CA, United States) and analyzed by Clampfit 10 software (Molecular Devices, Sunnyvale, CA, United States).

### *In vivo* Multi-Electrode Recording

We made a 32-channel micro-drive electrode according to previously described method (Lin et al., 2006; Ma et al., 2013). The 32-channel micro-drive electrode include plastic shaft, micro-drive system (screw and nut), one 36-pin connector (A79026-001, Omnetics, Minneapolis, MN, United States), a bundle of 8 pieces of polyimide tubing (inner diameter 100 μm, outer diameter 150 μm, Poly-microtechnologies, Phoenix, AZ, United States) glued to movable nuts. Every piece of polyimide tubing contains a tetrode wire. Each tetrode consisted of twisted four-electrode wires (12.5 μm, 90% platinum/10% iridium, #100-167, California Fine Wire, Grover Beach, CA, United States), securing four strands together with hot wind and removing insulation layer of the tip. Removing insulation layer tip of every wire electrode was sealed to 36-pin connector and the other side stretch out 3∼4mm beyond the end of polyimide tubing. Polyimide tubing and tetrode electrode wires were glued together. By turning the screw, nut moves in screw thread to move a bundle of polyimide tubing.

Wild-type or *PTEN* CKO mice were anesthetized with 60 mg/kg urethane and then were fixed in a stereotaxic apparatus (RWD Life Science Co., Ltd., Shenzhen, Guangdong, China) and eyes were coated with Erythromycin eye ointment. After removing hair above skull, betadine solution was smear on the skin surface and then 0.2 ml (2ml: 40mg) Procaine hydrochloride solution was injected under the skin surface. Scalpel cut along the midline of the skull and the membrane of skull was carefully removed by scissor to fully expose the skull. Cotton ball absorbed ethyl alcohol scrape the skull to remove the other tissue membrane in order to display the bregma and lambdoid suture that hold the same horizontal by adjusting stereotaxic apparatus. The 32-channel micro-drive electrode was implanted at CA1 of the dorsal hippocampus (2.2 mm posterior and 1.7 mm lateral to bregma). A small square skull surrounding the implanted place was drilled by dental drill and then dura was removed. This square hole was covered by the cotton ball absorbed saline. Four holes surrounding the square hole were drilled and small screws were installed every hole, one of four screws twined earth wire of electrode and then four screws were fixed with dental cement. Four screws were connected and heightened with dental cement to form a hollow wall surrounded square hole for electrode implantation. A bundle of 8 tetrodes of 32-channel micro-drive electrodes was lowered into the square hole, impaled the brain tissue and advanced 0.8 mm (brain surface was set as zero point). The gaps were filled with softened paraffin and the micro-drive electrode was connected to hollow wall of four screws by using dental cement. A copper net wrapped the micro-drive electrode. The mice were placed back to their home cage for recovery.

The mice recovered from surgery for 2–3 days and then were connected by Plexon Neural Data Acquisition System (Plexon Inc., Dallas, United States). The connector of electrode was connected head stage (20X amplification) of Plexon that was linked to pre-amplifiers with cables for recording neuronal signals of 32-channel in the tetrode format. Every day the screw of micro-drive electrode was turned to advance 1/6 cycle (50 μm) one time until the tip of tetrodes were slowly advanced into the hippocampal CA1 pyramidal layer through an identification of the field potential and neuronal activity patterns (Ylinen et al., 1995). The procedure of advancing electrode will take 5–10 days. After finding the good signal, neural firings (Plexon Inc., Dallas, United States) and movement trajectory (0.08 s/frame, Ethovision XT8, Noldus Information Technology, Wageningen, Netherlands) were recorded simultaneously during behavioral test. Continuous wide-band data of Plexon were imported into Plexon Offline Sorter software (Plexon Inc., Dallas, United States) for the spike detection. Units cluster was isolated and multi-unit spike timestamp in every tetrode channel was analyzed. Heatmaps were processed by custom MATLAB programs with the spike timestamp and corresponding to trajectory data.

### PTEN CKO in Dorsal CA1 With Adeno-Associated Virus

Adeno-associated virus construction and injection. The adeno-associated virus (AAV) construction (rAAV-CamkII-cre-WPRE-pA) was from Wuhan BrainVTA. The viruses (>10^12^ TU/ml) were used in the present study. For CA1 viral injections, *Pten*^*loxP/loxP*^ mice aged 3–4 weeks were anesthetized with pentobarbital (60 mg/kg, i.p.) and placed in a stereotaxic apparatus (RWD Life Science Co., Ltd., Shenzhen, Guangdong, China). Virus (0.3 μL in volume) was delivered to each hemisphere at AP 2 mm, ML 1.5 mm, DV 1.38 mm targeting for dorsal CA1. After each injection, the pipette was left in tissue for 10 min before slowly withdrawn, to prevent virus spilling over. Dental acrylic and cement were mixed and applied to connect the skull and plate for structural support. Mice were recovered for at least 3 weeks after the surgery. All the mice were sacrificed after experiments to confirm the injection sites and the viral trans-infection effects by using immunohistochemistry of cre and Pten.

### GO/NO GO Task

#### Behavioral Setups

We utilized a high-throughput automatic training system for odor-based learned behaviors in head-fixed mice, and trained mice to perform delayed paired association (DPA) following the paradigm described previously (Liu et al., 2014). Computer-controlled olfactometry systems were used for semi-automatic training. The microprocessor-based controller was used to switch on/off the solenoid valves for controlling water and odor delivery in millisecond temporal resolution. Three-way solenoid valves were used for controlling airflow, whereas two-way solenoid valves were used for controlling water flow. The total length of odor-delivery tubes (inner diameter: 2.5 mm; outer diameter: 4.0 mm) was minimized to increase the turnover rate of odorants. An exhaust tube connected to a vacuum pump was used to further minimize residual odor and suck out the water not licked in time for six behavioral setups were used. An independent controller was used for each behavioral setup. Odor and water supply and the connection to vacuum pump were also independent for all behavioral setups. All facility parameters, including length for all tubes and air-flow rate, were kept the same across all behavioral setups. Behavioral results from 4∼6 controllers were recorded simultaneously by custom-written software and stored in computers.

#### Behavioral Training

For the DPA task, a sample and a test odor were delivered, separated by a delay period (6 s). Four kinds of odorants were used, 1-Butanol (S1, Boiling point: 117.6 °C), Propyl formate (S2, Boiling point: 80–81°C), Propyl acetate (T1, Boiling point: 102 °C), 3-Methyl-2-buten-1-ol (T2, Boiling point: 140 °C). Odor delivery was monitored in real-time by alcohol sensors during the experiment. Odor delivery duration was set to 1 s, which was enough for rodents to perceive olfactory cues. The delay period between two odors in a trial was 6 s. The response window was set to 0.5–1 s after the offset of the test odor in a trial. Mice were trained to lick water reward only after the paired trials (S1-T1 or S2-T2). Licking events detected in the response window in paired trials were regarded as Hit and will trigger instantaneous water delivery. In hit trials water was provided at a speed of ∼1.7 mL/min for half a second in a response time window. The false choice was defined as detection of licking events in the response window in non-paired trials (S1-T2 or S2-T1), and mice were not punished in False Choice trials. Mice were neither punished nor rewarded for Miss (no-lick in the paired trial) or CR (no-lick in a non-paired trial) trials. In each day, mice were required to perform 168 trials. Behavioral results were grouped in sessions of 24 trials. There was no break between sessions, i.e., session was used for convenience in presenting behavioral results. There was a fixed inter-trial interval of 12 s between trials unless stated otherwise. After training sessions ended each day, mice were supplied with free water until satiety. This training phase lasts for 5 consecutive days. The well-trained criterion was set to the existence of three continuous correct rates larger than 80%, calculated using a session of 24 trials.

Before the start of training, mice were water restricted for 1 day. The behavioral training process included habituation, shaping, and DPA learning phases. In habituation phase, mice were head-fixed in behavioral setups and trained to lick water from a water tube, encouraged with manually delivered water through syringes (5 mL). Tips of syringe needles (size of 27G) were cut and polished to ensure safety. Typically, in 2 days, mice could learn to lick for 1 to 2 min without manual water delivery. The shaping phase was then started, in which only paired trials were applied and water was provided in all 168 trials each day. At the beginning of shaping phase, water was delivered manually through syringes to encourage mice to lick in the response window. However, manual water delivery was temporally withheld to check whether mice could lick in the response window spontaneously. Shaping phase ended once mice could lick for water without manual delivery for consecutive 24 trials (one session). Typically, the shaping phase lasted for 3 days.

The DPA learning phase was then started from the next day, which was defined as Day 1 in the behavioral analysis reported in all figures. Both paired and non-paired trials were applied pseudo-randomly, i.e., two non-paired and two paired trials of balanced odor-pairs were presented randomly in every consecutive four trials. No human intervene was applied in the DPA learning phase to minimize any potential human bias in behavioral results. Typically, behavioral performance was visualized in sessions.

#### Data Analysis

The performance of the correct rate (referred to as “performance” in labels of figures) of each session was defined by:

Performance correct rate = (num. hit trials + num. correct rejection trials)/total number of trials.

Hit, False choice and CR rates were defined as follows:

Hit rate = num. hit trials/ (num. hit trials + num. miss trials)

correct rejection rate = num. correct rejection trials/ (num. flase chose trials + num. correct rejection trials)

### Statistical Analysis

Statistical analyses were performed by using two-tailed Student’s t test or one-way or two-way ANOVA followed by Tukey’s or Sidak’s *post hoc* test when the data pass normality test. For comparison of groups that are not normally distributed, non-parametric unpaired Mann-Whitney’s non-parametric *U* test, paired Wilcoxon test, Kolmogorov-Smirnov test, Kruskal–Wallis’s one-way ANOVA and Friedman’s two-way ANOVA tests followed by a post-hoc multiple comparison Dunn’s test. Normality test was assessed by using D’Agostino–Pearson’s test and Shapiro–Wilk test. All the tests were conducted in GraphPad Prism 9 and Origin 9.

## Results

### Short-Term/Long-Term Memory for Social Recognition

**FIGURE 1:**
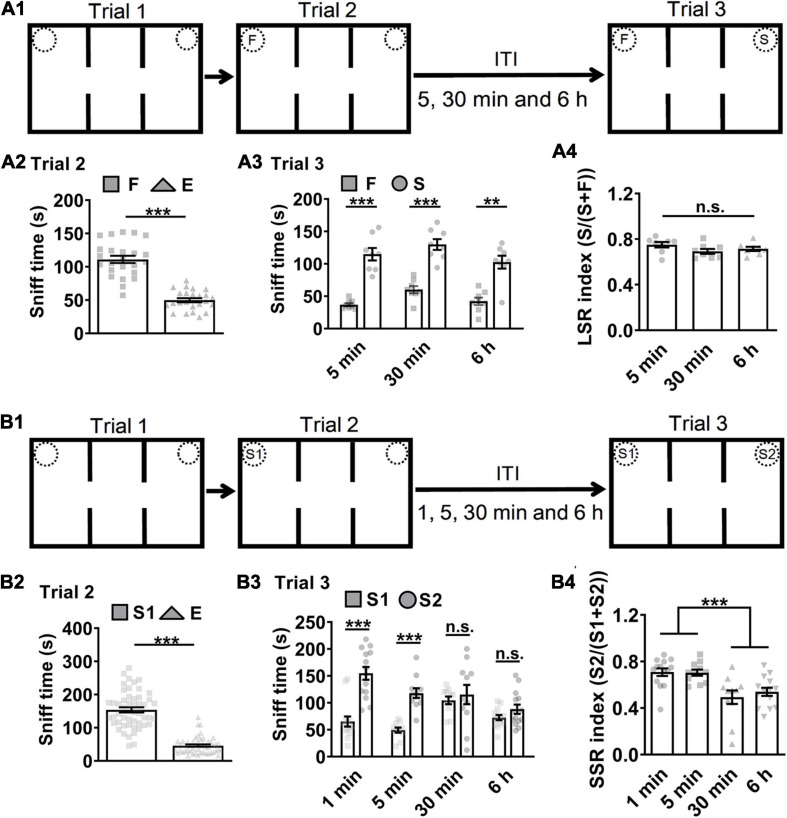
Mice short-term memory monitored in social recognition test. **(A1)** Schematic of experimental paradigm. ITI, inter-trial intervals; F, a familiar littermate of the subject mice. S, a stranger mouse. **(A2)** Subject mice exhibited preference (more sniff time) to F than E, suggesting normal sociability in trial 2 by F *vs.* E (*n* = 24; *t* = 11.770, ***P < 0.0001). **(A3)** Subject mice showed preference to S than F in trial 3 (*n* = 8 for ITI 5 min, *t* = 7.832, *P* < 0.0001; *n* = 8 for ITI 30 min, *t* = 7.002, *P* < 0.0001; *n* = 8 for ITI 6 h, Wilcoxon test, *P* = 0.0078). **(A4)** Subject mice showed higher LSR index to S than F in trial 3 by F *vs.* S regardless of the ITIs (*n* = 8 for 5 min; *n* = 8 for 30 min; *n* = 8 for 6 h; *F*_(2,21)_ = 1.903, *P* = 0.1739), suggesting that long-term memory for F can guide social recognition. **(B1)** Schematic of experimental paradigm. S1, a first stranger mouse; S2, a second stranger mouse; E, empty cage. **(B2)** Subject mice exhibited preference to S1 over E, suggesting normal sociability in trial 2 by S1 *vs.* E (*n* = 50; Wilcoxon test, *P* < 0.0001). **(B3)** Subject mice showed preference to S2 over S1 under the ITIs for 1 or 5 min but not for 30 min or 6 h (*n* = 14 for 1 min, Wilcoxon test, *P* = 0.0006; *n* = 11 for 5 min, *t* = 6.699, *P* < 0.0001; *n* = 11 for 30 min, *t* = 0.5549, *P* = 0.5851; *n* = 14 for 6 h, *t* = 1.574, *P* = 0.2388). **(B4)** Subject mice showed higher SSR index to S2 over S1 under the ITIs for 1 or 5 min but not for 30 min or 6 h, suggesting that normal social recognition in trial 3 by S1 *vs.* S2 was limited by social short-term memory for experienced S1 under 30 min (*n* = 14 for 1 min; *n* = 11 for 5 min; *n* = 11 for 30 min; *n* = 14 for 6 h; *P* = 0.0002, χ2 = 20.13. Dunn’s multiple comparisons test, 1 min vs. 5min, *P* > 0.9999; 1 min vs. 30 min, *P* = 0.0042;1 min vs. 6h, *P* = 0.0054; 5 min vs. 30 min, *P* = 0.0163; 5 min vs. 6h, *P* = 0.0228; 30 min vs.6h, *P* > 0.9999.). ***P < 0.001; n.s., not significant. Data presented as mean ± SEM. Statistical analysis was performed by using Wilcoxon test, Kruskal–Wallis test followed by Dunn’s *post hoc* analysis, student’s *t* test or one-way ANOVA followed by Tukey’s *post hoc* analysis.

A three-chamber and three-trial social interaction test was widely adopted for evaluation of sociability and social recognition in mice (Moy et al., 2004; Etherton et al., 2011; Peca et al., 2011; Han et al., 2012; Gkogkas et al., 2014; Cao et al., 2018b). Trial 1 examined subject mice’s response to environmental novelty by allowing them to explore the three-chamber with a left empty cage (E) *vs.* a right empty cage (E). Trial 2 tested subject mice’s sociability to a familiar littermate (F) *vs.* an empty cage (E) (Figure 1A1). Subject mice’s sociability in trial 2 by exploring F *vs.* E was indicated by more sniff time to F than to E (Figure 1A2, ^∗∗∗^*P* < 0.001). Trial 3 was for social recognition test during which subject mice tend to explored more often with a novel stranger mouse (S) *vs.* F while holding the memory of F. To test whether the memory of F was LTM which typically last more than several hours, then we enhanced memory load by increasing inter-trial intervals (ITI) from 5, 30 min to 6 h and accordingly assigned subject mice randomly into three independent groups. Subject mice’s social recognition in trial 3 by exploring F *vs.* S was indicated by more sniff time to S than to F (Figure 1A3, ^∗∗∗^*P* < 0.001). LTM-guided social recognition index (LSR index) was calculated for group comparison purpose. Here, the LSR index was similar among ITI 5, 30 min and 6 h (Figure 1A4).

After similar trial 1 as above (Figure 1B1), subject mice were allowed to explore a first stranger mouse in the E (S1) *vs.* E in trial 2 for 10 min during which subject mice could form social memory about S1. Subject mice’s sociability in trial 2 by exploring S1 *vs.* E was indicated by more sniff time to S1 than to E (Figure 1B2, ^∗∗∗^*P* < 0.001). To test whether the memory of S1 was STM or LTM, then we designed the inter-trial intervals (ITI) for 1, 5, 30 min, or 6 h and accordingly assigned subject mice randomly into four independent groups before the starting of trial 3 for social recognition test, during which subject mice explored S1 *vs.* a second stranger mouse (S2). S1 and S2 were age- and gender-matched conspecifics from different batches and housed separately. Subject mice’s social recognition in trial 3 by exploring S1 *vs.* S2 as indicated by more sniff time or higher social indexes to S2 than to S1 was highly significant if the ITIs were set at a short interval, 1 or 5 min (Figure 1B3, ^∗∗∗^*P* < 0.001), but not at a longer one, 30 min or 6 h (*P* > 0.05). STM-guided social recognition index (SSR index) was calculated for group comparison purpose. SSR indexes also demonstrated significant difference between the short and long ITIs (Figure 1B4, ^∗∗∗^*P* < 0.001). The locomotor activity in ITI 1 min group was significantly higher which may contribute to higher SSR index. However, the locomotor activity in ITI 5 min group was similar as ITI 30 min and 6 h groups (Supplementary Figure 7A). Thus, the ITI 5 min was chosen for the future social recognition test unless stated otherwise. To test whether the increased exploration of the new stranger mouse relied on the memory that one cup was empty in trial 2 and now occupied in trial 3, a familiar littermate (F) (Supplementary Figure 1A1) or a mouse-size object (Ob) (Supplementary Figure 1B1) instead of S2 was enclosed in the previously empty cup after normal sociability test in trial 2 (Supplementary Figures 1A2, 1B2, ^∗∗∗^*P* < 0.001). However, there was no preference by exploring S *vs.* F (Supplementary Figure 1A3) or even preference for S *vs.* Ob (Supplementary Figure 1B3, ^∗^*P* < 0.05) in trial 3. Thus, we argued that the increased exploration of the new stranger mouse required that the subject mice distinguished between F and S or S1 and S2 (i.e., social memory). In addition, since social recognition was susceptible to ITI 30 min or 6 h interference, we suggest that STM formation of S1 was attributable.

### Optogenetic Silencing of Dorsal CA1 Neuronal Activity

**FIGURE 2:**
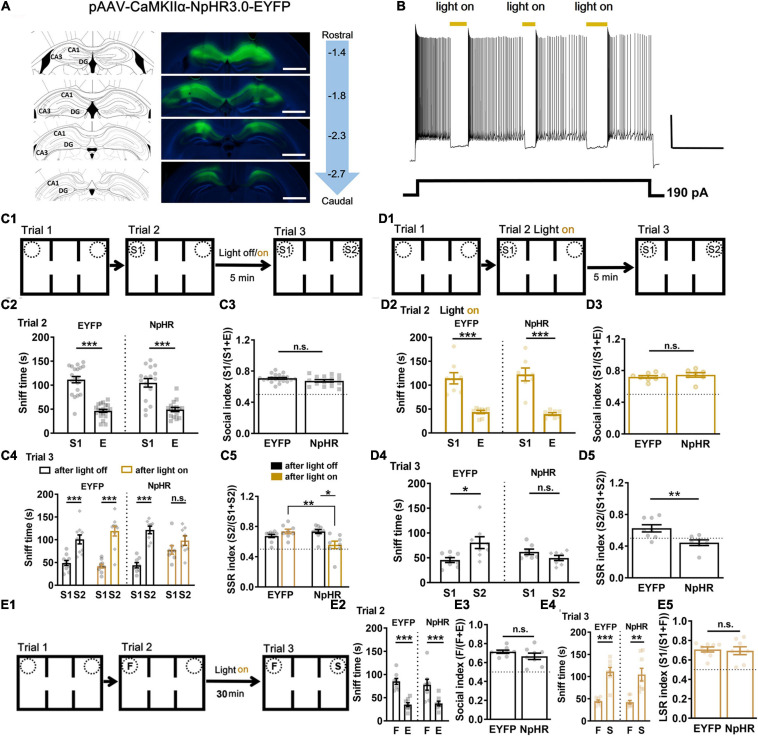
Short-term but not long-term social memory-guided recognition disrupted by optogenetic silencing of CA1 neuronal activity. **(A)** Expression of pAAV-CaMKII-NpHR3.0-EYFP and pAAV-CaMKII-EYFP was checked in CA1 regions of the dorsal hippocampus at 3 weeks after the virus injection. Left: injection site according to the mouse brain atlas. Right: viral expression in CA1 regions, and distance (in mm) from bregma on the sagittal suture. Calibration bars, 1 mm. **(B)** Action potentials elicited by current injection were inhibited by light on (yellow, 594 nm) in NpHR3.0 expressed CA1 neurons *in vitro*. Calibration bar, horizontal 10 s, vertical 20 mV. **(C1)** Schematic of experimental paradigm for SSR. Light was given during ITI 5 min. **(C2)** All the mice showed normal sociability in trial 2 by exploring S1 vs. E (*n* = 20 for EYFP, *t* = 8.8717, *P* < 0.0001; *n* = 16 for NpHR, *t* = 5.56007, *P* < 0.0001). **(C3)** All groups showed normal sociability with regards to social index in trial 2 (*n* = 20 for EYFP, *n* = 16 for NpHR, *t* = 1.696, *P* = 0.0990). **(C4)** Light on during the 5-min ITI impaired subsequent social recognition in trial 3 by exploring S1 vs. S2 in NpHR group but not in EYFP group, while light off had no significant effect on both the groups (*n* = 10 for EYFP with light off, *t* = 4.56881, *P* = 0.000476; *n* = 10 for EYFP with light on, *t* = 6.69973, *P* < 0.0001; *n* = 8 for NpHR with light off, *t* = 7.6989, *P* < 0.0001; *n* = 8 for NpHR with light on, Wilcoxon test, *P* = 0.1953). **(C5)** NpHR with light on group showed lower SSR index in contrast to EYFP with light group or to NpHR with light off group (*n* = 10 for EYFP, *n* = 8 for NpHR; *P* = 0.0027, χ2 = 14.18. Dunn’s multiple comparisons test, EYFP on vs. NpHR on, *P* = 0.0036; NpHR off vs. NpHR on, *P* = 0.0245; ***P < 0.001). n.s., not significant. **(D1)** Schematic of experimental paradigm for SSR. Light was given during trial 2. **(D2)** All the mice showed normal sociability in trial 2 by exploring S1 vs. E (*n* = 8 for EYFP, *t* = 5.7557, *P* < 0.0001; *n* = 7 for NpHR, *t* = 5.9880, *P* < 0.0001). **(D3)** Light on during trial 2 by exploring S1 vs. E had no significant effects on sociability in terms of social index (*n* = 8 for EYFP, *n* = 7 for NpHR, *t* = 0.7935, *P* = 0.4417). **(D4)** Light on during trial 2 still impaired social recognition in trial 3 by exploring S1 vs. S2 in NpHR but not EYPF control (*n* = 8 for EYFP, Wilcoxon test, *P* = 0.0391; *n* = 7 for NpHR, *t* = 1.6203, *P* = 0.1311; *P < 0.05). **(D5)** NpHR with light on group showed lower SSR index in contrast to EYFP with light group (*n* = 8 for EYFP, *n* = 7 for NpHR, *t* = 3.076, *P* = 0.0089; **P < 0.01). **(E1)** Schematic of experimental paradigm for LSR. **(E2)** All the mice showed normal sociability in trial 2 by exploring F vs. E (*n* = 8 for EYFP, *t* = 4.7343, *P* = 0.000115; *n* = 8 for NpHR, *t* = 3.8325, *P* = 0.000657; ***P < 0.001). **(E3)** EYFP and NpHR groups showed similar sociability with regards to social index in trial 2 (*n* = 8 for EYFP, *n* = 8 for NpHR, *t* = 1.269, *P* = 0.2252). **(E4)** Light on during the 30-min ITI did not affect social recognition in trial 3 by exploring F vs. S in NpHR group (*n* = 8 for EYFP, *t* = 6.965, *P* = 0.000437; *n* = 8 for NpHR, *t* = 3.688, *P* = 0.007781; **P < 0.01; ***P < 0.001). **(E5)** NpHR with light on group showed similar LSR index as EYFP group (*n* = 8 for EYFP, *n* = 8 for NpHR, *t* = 0.2723, *P* = 0.7894). S1, a first stranger mouse; E, empty cage; S2, a second stranger mouse; F, a familiar littermate of the subject mice. Data presented as mean ± SEM. Statistical analysis was performed by using Wilcoxon test, Kruskal–Wallis test followed by Dunn’s *post hoc* analysis, student’s *t* test or two-way ANOVA followed by Sidak’s *post hoc* analysis.

During social interaction, spatial representations in STM are crucial contexts allowing binding of social features that belongs together with STM. Did spatial representation for which dorsal hippocampal CA1 was responsible play a role in memory-guided social recognition? Spatial position swap interference (F’s location exchanged with S’s in trial 3, Supplementary Figure 1C1) did not affect social recognition in trial 3 (Supplementary Figure 1C3, ^∗∗∗^*P* < 0.001) when subject mice explored F *vs.* E in trial 2 (Supplementary Figure 1C2, ^∗∗∗^*P* < 0.001). When S1’s location exchanged with S2’s in trial 3 (Supplementary Figure 1D1), subject mice explored more often with S1 in trial 2 (Supplementary Figure 1D2) and still more often with S2 in trial 3 (Supplementary Figure 1D3). In order to test whether dorsal hippocampal CA1 neuronal activity was necessary for STM in organization of social recognition, we used optogenetic tool AAV-CaMKIIα-NpHR3.0-EYFP while AAV-CaMKIIα-EYFP as control (Figure 2A). NpHR was confirmed by using a pulse of yellow light (594 nm) *via* an optical fiber to have suppressed the evoked neuronal firing *in vitro* (Figure 2B). Then, these mice were subjected to STM-guided social behavioral study (Figure 2C1). Both NpHR and EYFP groups showed normal sociability (S1 *vs.* E) in trial 2 (Figures 2C2,C3, ^∗∗∗^*P* < 0.001). Because we wanted to test whether STM formed during sociability test can endow subsequent social recognition, we first gave three pulses of yellow light (light on, 1 min duration and 1 min interval) to half of the subject mice during the ITIs for 5 min in the middle chamber before trial 3, while the other half was set as light off. One week later, half of light on were treated as light off, and *vice versa*. All data were pulled together for analysis. We found that optogenetic silencing of dorsal CA1 neuronal activity during the ITIs for 5 min disrupted subsequent social recognition (S1 *vs.* S2) in NpHR group but not in EYFP control, while light off had no significant effect on both the groups (Figures 2C4,C5, ^∗∗∗^*P* < 0.001). The locomotor activity was not significantly different among groups (Supplementary Figure 7B). Thereafter, in independent groups (Figure 2D1), a constant yellow light (594 nm) was turned on/off manually once the nose of subject mice got in/out the pre-defined sniff zone within 2 cm proximity of the cylindrical cage confining S1, we found that light on during trial 2 (S1 *vs.* E) had no significant effect on sociability test (Figures 2D2,D3, ^∗∗∗^*P* < 0.001), but that still led to impaired social recognition (S1 *vs.* S2) in trial 3 in NpHR group but not in EYFP control (Figures 2D4,D5, ^∗∗^*P* < 0.01). The locomotor activity was not significantly different between two groups (Supplementary Figure 7C). Furthermore, optogenetic silencing of dorsal CA1 neuronal activity during the ITIs for 30 min (15 pulses of yellow light, light on, 1 min duration and 1 min interval, Figure 2E1) did not affect sociability (Figures 2E2,E3) and social recognition (F *vs*. S) in both NpHR and EYFP groups (Figures 2E4,E5). Thus, these findings strongly supported that, rather than for sociability and/or LTM, dorsal CA1 neuronal activity was critical for STM, which might enable the subject mice to hold the information temporally about S1 and relevant context under 30 min so that the subject mice preferred S2 over S1 in social recognition test.

### CA1 PTEN KD in Social Recognition

**FIGURE 3:**
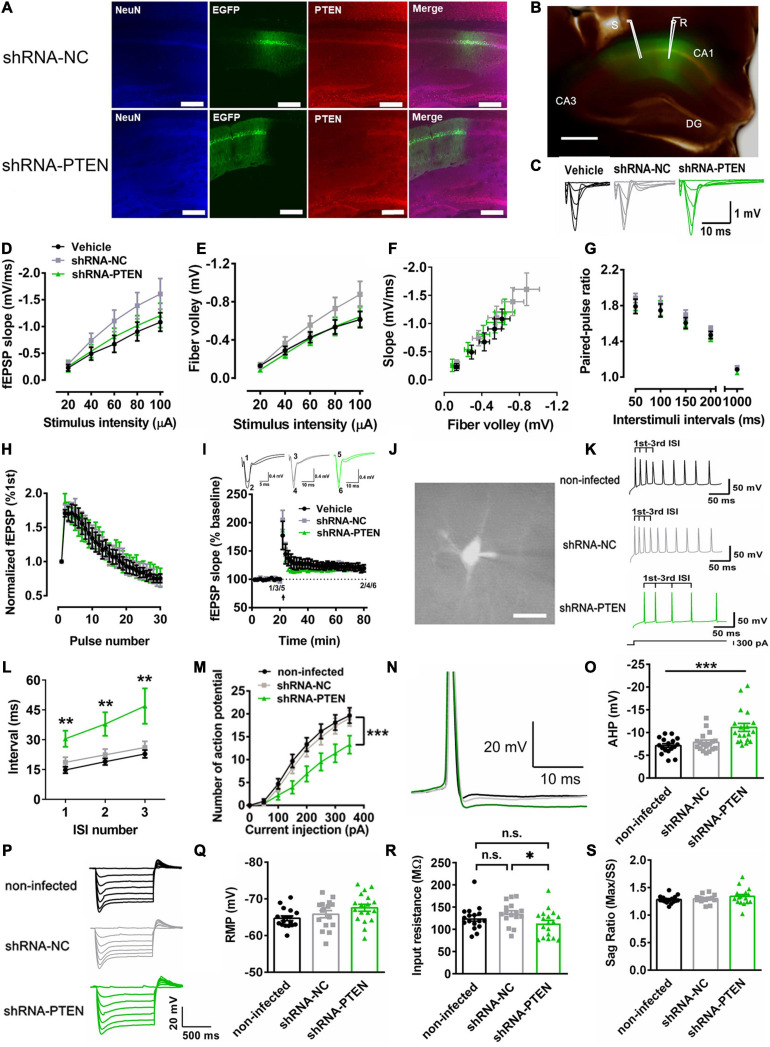
Reduced firing rate *in vitro* with CA1 PTEN knock down (KD). **(A)** Immunofluorescence staining of NeuN and PTEN on lentivirus-infected dorsal CA1 cells labeled with EGFP. In LV-shRNA-PTEN-GFP group, cells labeled with EGFP were not labeled with anti-PTEN antibody. Calibration bar, 150 μm. **(B)** Merged bright-field with EGFP fluorescence image showed representative fEPSP recording in virus-affected CA1 region of the dorsal hippocampus by stimulating the Schaffer collaterals in brain slices. Calibration bar, 500 μm. **(C)** Traces for fEPSP recordings of input-output curve. **(D)** The input-output curve plotted with stimulus intensity against slope was not significant difference among the groups (*n* = 8 from 3 mice for Vehicle, *n* = 7 from 3 mice for the other two groups; Two-way ANOVA with Sidak’s *post hoc* test. Factor of stimulus intensity x treatment, *F*_(8,76)_ = 1.956, *P* = 0.0636; Factor of stimulus, *F*_(4,76)_ = 99.92, *P* < 0.0001; Factor of treatment, *F*_(2, 19)_ = 1.338, *P* = 0.2860; *P* > 0.05). **(E)** The input-output curve plotted with stimulus intensity against fiber volley was not significant difference among the groups (*n* = 8 from 3 mice for Vehicle, *n* = 7 from 3 mice for the other two groups; Two-way ANOVA with Sidak’s *post hoc* test. Factor of stimulus intensity x treatment, *F*_(8, 76)_ = 1.994, *P* = 0.0584; Factor of stimulus, *F*_(4, 76)_ = 124.0, *P* < 0.0001; Factor of treatment, *F*_(2, 19)_ = 1.326, *P* = 0.2890; *P* > 0.05). **(F)** The input-output curve plotted with slope against fiber volley. **(G)** A form of short-term synaptic plasticity, the paired-pulse facilitation, was not significant different among the groups (*n* = 8 from 3 mice for Vehicle, *n* = 7 from 3 mice for the other two groups, *P* > 0.05). **(H)** Another form of short-term synaptic plasticity, the short-term augmentation at 20 Hz, was also not significant different among the groups (*n* = 8 from 3 mice for Vehicle, *n* = 7 from 3 mice for the other two groups, *P* > 0.05). **(I)** Long-term potentiation was not significant different among the groups (*n* = 6 from 3 mice for Vehicle, *n* = 7 from 3 mice for the other two groups, *P* > 0.05). **(J)** Whole-cell recordings via the fluorescence of CA1 neurons. Calibration bar, 20 μm. **(K)** Examples of voltage trace elicited by a constant current injection for 1 s at 300 pA. **(L)** The first three inter-spike intervals (ISIs) were monitored. The shRNA-PTEN group showed higher ISI in contrast to non-infected and shRNA-NC groups (*n* = 17 for non-infected from 5 mice, *n* = 18 for shRNA-NC from 4 mice, *n* = 18 for shRNA-PTEN from 6 mice; 1st, *P* = 0.0037. χ2 = 11.18. 1st Vehicle vs. 1st shRNA-NC, *P* = 0.9594; 1st Vehicle vs. 1st shRNA-PTEN,*P* = 0.0035; 1st shRNA-NC vs. 1st shRNA-PTEN, *P* = 0.0664; 2nd, *P* = 0.0061. χ2 = 10.20. 2nd Vehicle vs. 2nd shRNA-NC, *P* > 0.9999; 2nd Vehicle vs. 2nd shRNA-PTEN,*P* = 0.0081; 2nd shRNA-NC vs. 2nd shRNA-PTEN, *P* = 0.0448; 3rd, *P* = 0.0059. χ2 = 10.26. 3rd Vehicle vs. 3rd shRNA-NC, *P* > 0.9999; 3rd Vehicle vs. 3rd shRNA-PTEN,*P* = 0.0096; 3rd shRNA-NC vs. 3rd shRNA-PTEN, *P* = 0.0326). **(M)** The firing rate of the evoked action potentials was significantly reduced in shRNA-PTEN neurons compared with NC or non-infected neurons (*n* = 17 for non-infected from 5 mice, *n* = 18 for shRNA-NC from 4 mice, *n* = 18 for shRNA-PTEN from 6 mice; ^∗∗∗^*P* < 0.001). **(N)** Sample traces of after-hyperpolarization (AHP) of action potentials elicited by a constant current injection for 1 s at 300 pA. **(O)** Increased AHP at 25 ms after the action potential onset in shRNA-PTEN neurons compared with NC and non-infected neurons (*n* = 17 for non-infected from 5 mice, *n* = 18 for shRNA-NC from 4 mice, *n* = 18 for shRNA-PTEN from 6 mice; *P* = 0.0002. χ2 = 17.59. non-infected vs. shRNA-NC, *P* > 0.9999; non-infected vs. shRNA-PTEN,*P* = 0.0004; shRNA-NC vs. shRNA-PTEN, *P* = 0.0027). **(P)** Representative response of voltage steps by injecting hyperpolarization current from resting membrane potential. **(Q)** No significant difference in resting membrane potential (RMP) among the groups (*n* = 18 for shRNA-PTEN from 6 mice, *n* = 17 for non-infected from 5 mice, *n* = 16 for shRNA-NC from 4 mice, *P* > 0.05). **(R)** Input resistance was significantly different among the groups (*n* = 18 for shRNA-PTEN from 6 mice, *n* = 17 for non-infected from 5 mice, *n* = 16 for shRNA-NC from 4 mice; *P* = 0.0368, χ2 = 6.602. Dunn’s multiple comparisons test, non-infected vs. shRNA-NC, *P* = 0.4762; non-infected vs. shRNA-PTEN, *P* = 0.7410; shRNA-NC vs. shRNA-PTEN, *P* = 0.0307). **(S)** No sag ratio difference among the groups (*n* = 18 for shRNA-PTEN from 6 mice, *n* = 17 for non-infected from 5 mice, *n* = 16 for shRNA-NC from 4 mice, *F*_(2, 48)_ = 1.392, *P* = 0.2585). Data presented as mean ± SEM. Statistical analysis was performed by using Kruskal–Wallis test followed by Dunn’s *post hoc* analysis and two-way or one-way ANOVA followed by Tukey’s *post hoc* analysis.

To further confirm our idea that dorsal CA1 neuronal activity may underlie social STM for social recognition, we then used PTEN mouse model of ASD. Previous study has demonstrated that PTEN loss in cortical neurons lead to neuronal hypoactivity (Garcia-Junco-Clemente et al., 2013). Thus, lentivirus (LV) [LV-shRNA-PTEN-EGFP or LV-shRNA-NC-EGFP as a negative control (NC)] to express PTEN-shRNA for knockdown (KD) was injected in the dorsal CA1 region of the dorsal hippocampus. Three weeks after the injection of the viruses, there was a marked reduction in immunoreactivity of PTEN in neurons infected with LV-shRNA-PTEN-EGFP compared with that with LV-shRNA-NC-EGFP control (Figure 3A). Merged bright field and GFP image showed robust expression of EGFP in dorsal hippocampal CA1 and subsequent field excitatory postsynaptic potential (fEPSP) recordings at Schaffer-collateral (SC)-CA1 pathway (Figure 3B). Change of basal synaptic transmission and synaptic plasticity could significantly interfere with cognitive functions that are known to depend on hippocampus such as learning and memory (Patterson et al., 1996; Citri and Malenka, 2008; Neves et al., 2008). The response of fEPSP was stimulated at various stimulus intensities (Figure 3C). We found no significant differences among the groups in input-output curves for testing basal synaptic weight in terms of fEPSP slope (Figure 3D), presynaptic fiber volley (Figure 3E) and function relating fiber volley amplitude to fEPSP slope (Figure 3F). These results indicated that basal synaptic transmission was unaltered in dorsal hippocampal SC-CA1 pathway with PTEN deletion. Short-term synaptic plasticity is usually attributed to presynaptic residual calcium elevation last for at most a few minutes (Zucker and Regehr, 2002). It has been reported that short-term synaptic plasticity may underlie working memory (Mongillo et al., 2008). Here, two major forms of short-term synaptic plasticity, paired-pulse facilitation (Figure 3G) and short-term augmentation at 20 Hz (Figure 3H) were tested. No significant difference were found among PTEN KD and control groups, suggesting normal short-term plasticity in dorsal hippocampal SC-CA1 pathway with PTEN deletion. Besides, while it is well-known that synaptic LTP represents a biological substrate for LTM (Takeuchi et al., 2014), LTP was not significantly altered after PTEN deletion in SC-CA1 pathway (Figure 3I). Furthermore, changes in neuronal excitability has been linked to STM and WM (Fuster and Alexander, 1971; Constantinidis et al., 2018). Therefore, the role of PTEN in regulating neuronal excitability was studied. In the whole-cell recordings (Figure 3J), we found that shRNA-PTEN group as compared with NC and vehicle controls exhibited significantly higher level of spike frequency adaptation (Figures 3K,L, ^∗∗^*P* < 0.001, ^∗^*P* < 0.05) and lower rate in firing action potentials (Figure 3M, ^∗∗∗^*P* < 0.001), likely due to significantly larger after-hyperpolarization (AHP) currents (Figures 3N,O, ^∗∗∗^*P* < 0.001), possibly consistent with previous report (Benda and Herz, 2003; Garcia-Junco-Clemente et al., 2013). In order to investigate whether neuronal hypoactivity was contributed by potential changes in passive membrane properties and hyperpolarization-activated currents due to PTEN deletion (Albertson et al., 2011; Tamagnini et al., 2015), the other sub-threshold intrinsic membrane properties such as the response to hyperpolarizing current injection (Figure 3P), resting membrane potential (Figure 3Q), input resistance (Figure 3R) and sag ratio (Figure 3S) were tested. It was shown that input resistance was significantly lower in shRNA-PTEN group compared to that in shRNA-NC group, the others are not significantly different among the groups. To sum, postnatal PTEN loss in CA1 neurons of dorsal hippocampus led to lower neuronal activity which correlated with STM and WM without affecting basal synaptic transmission and plasticity which pertained to LTM.

**FIGURE 4:**
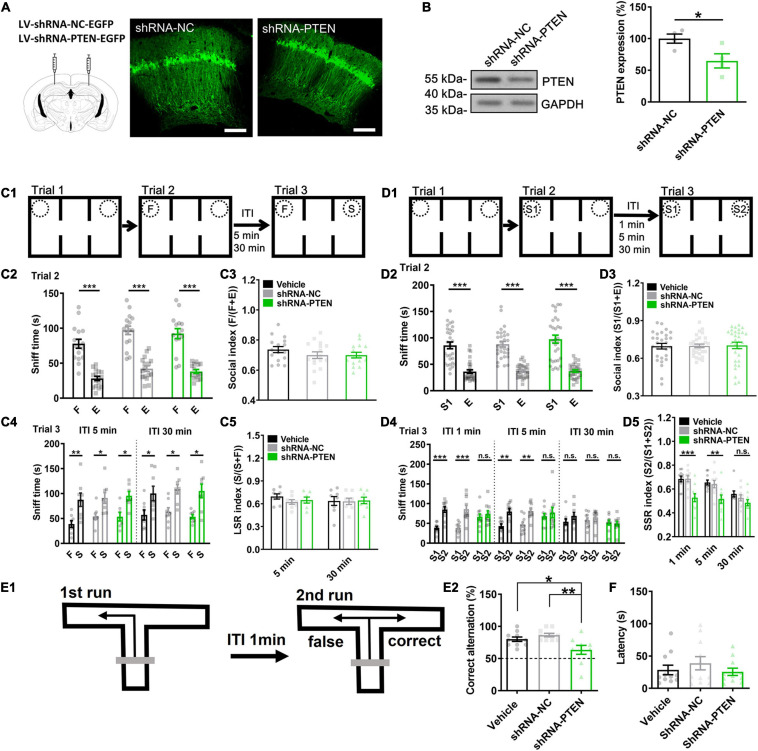
Short-term memory-guided social recognition deficit in CA1 PTEN knock down (KD) mice. **(A)** Juvenile mice were injected with lentivirus (LV) containing shRNA-NC-EGFP (negative control) or shRNA-PTEN-EGFP in dorsal CA1 regions. Three weeks after injection, robust EGFP was expressed in CA1 neurons indicating successful infection. Calibration bars, 200 μm. **(B)** Western blot of the EGFP expressed CA1 samples showed significant PTEN KD (*n* = 4/group; *P < 0.05). **(C1)** Schematic of experimental paradigm for LSR. **(C2)** All the mice explored more with F vs. E in trial 2. **(C3)** All groups showed normal sociability in trial 2 by exploring F vs. E (*n* = 16 for Vehicle, *n* = 16 for shRNA-NC, *n* = 15 for shRNA-PTEN; *F*_(2.44)_ = 1.102, *P* = 0.3412). **(C4)** All groups explored more with S vs. F regardless of the ITIs in trial 3(ITI 5 min, Vehicle, *t* = 4.468, *P* = 0.0029; shRNA-NC, *t* = 3.235, *P* = 0.0143*;* shRNA-PTEN, *t* = 3.340, *P* = 0.0156; ITI 30 min, Vehicle, *t* = 2.438, *P* = 0.0287; shRNA-NC, *t* = 3.172, *P* = 0.0157*;* shRNA-PTEN, *t* = 3.30, *P* = 0.0131; *P < 0.05; **P < 0.01). **(C5)** All groups expressed normal LSR index regardless of the ITIs (*n* = 8/group, but *n* = 7 for shRNA-PTEN group with the ITI for 5 min. ITI 5 min, *F*_(2_,_20)_ = 1.189, *P* = 0.3253. ITI 30 min, *F*_(2_,_21)_ = 0.01747, *P* = 0.9827). **(D1)** Schematic of experimental paradigm for SSR. **(D2)** All the mice explored more with S1 vs. E in trial 2. **(D3)** Sociability in trial 2 by exploring S1 vs. E was intact in all groups (*n* = 29 for Vehicle, *n* = 30 for shRNA-NC, *n* = 30 for shRNA-PTEN, *F*_(2.86)_ = 0.02262, *P* = 0.9776). **(D4)** The shRNA-PTEN group explored with S2 vs. S1 with similar time in ITI 1 and 5 min compared to that in Vehicle (0.01 M PBS) or shRNA-NC group. All group explored with S2 vs. S1 similarly in ITI 30 min. **(D5)** SSR index in trial 3 by exploring S1 vs. S2 was impaired in shRNA-PTEN group with the ITI for 1- or 5-min (ITI 1 min, *F*_(2,27)_ = 10.82, *P* = 0.0004. Tukey’s *post hoc* test. Vehicle vs. shRNA-NC, *P* = 0.9984; Vehicle vs. shRNA-PTEN, *P* = 0.0012; shRNA-NC vs. shRNA-PTEN, *P* = 0.0011. ITI 5 min, *F*_(2,27)_ = 6.357, *P* = 0.0039. Tukey’s *post hoc* test. Vehicle vs. shRNA-NC, *P* = 0.9417; Vehicle vs. shRNA-PTEN, *P* = 0.0064; shRNA-NC vs. shRNA-PTEN, *P* = 0.0143; **P < 0.01; ***P < 0.001). Social recognition in trial 3 by exploring S1 vs. S2 was absent in all groups with the ITI for 30 min (*n* = 10/group, but *n* = 9 in Vehicle with ITI for 30 min. *P* = 0.1261, χ2 = 4.141. Dunn’s multiple comparisons test, 30 min Vehicle vs. 30 min shRNA-NC, *P* > 0.9999; 30 min Vehicle vs. 30 min shRNA-PTEN,*P* = 0.1422; 30 min shRNA-NC vs. 30 min shRNA-PTEN, *P* = 0.5160; **P < 0.01; ***P < 0.001). n.s., not significant. S, a stranger mouse; S1, a first stranger mouse; S2, a second stranger mouse; E, empty cage; F, a littermate of the subject mice. **(E1)** Experimental paradigm of T-maze spontaneous alternation test. The test contains two phases. In the sample phase (the first run), mouse is started from the base of the T and allowed to choose and enter one of the goal arms. The mouse was then returned to the base and kept for 1-min ITI. In the choice phase (the second run), the mouse tends to choose the unsampled arm, reflecting correct memory of the first choice. **(E2)** The correct rate in 14 tests in total was significantly lower in shRNA-PTEN group compared to that in Vehicle (0.01 M PBS) or shRNA-NC group (*n* = 10 for Vehicle, *n* = 10 for shRNA-NC, *n* = 9 for shRNA-PTEN. *F*_(2,26)_ = 6.587, *P* = 0.0049. Tukey’s *post hoc* test. Vehicle vs. shRNA-NC, *P* = 0.5717; Vehicle vs. shRNA-PTEN, *P* = 0.0437; shRNA-NC vs. shRNA-PTEN, *P* = 0.0042; *P < 0.05; **P < 0.01). **(F)** Normal olfaction test in CA1 PTEN KD mice was observed compared to NC and Vehicle controls (*n* = 10/group; *P* = 0.8948. χ2 = 0.2224. Dunn’s multiple comparisons test, Vehicle vs. shRNA-NC, *P* > 0.9999; Vehicle vs. shRNA-PTEN,*P* > 0.9999; shRNA-NC vs. shRNA-PTEN, *P* > 0.9999; *P* > 0.05). Data presented as mean ± SEM. Statistical analysis was performed by using Mann Whitney’s *U* test, Kruskal–Wallis test followed by Dunn’s *post hoc* analysis, student’s *t* test or one-way ANOVA followed by Tukey’s *post hoc* analysis.

Consequently, the role of PTEN of CA1 neurons of dorsal hippocampus in regulating social cognition and memory was investigated during postnatal stage. Three weeks after injection, lentivirus transfection was mostly restricted in dorsal CA1 indicated by locally expressed EGFP (Figure 4A). Then, we quantified the KD efficiency by using western blot on the EGFP expressed CA1 samples. There was about 35.1 ± 9.9% reduction of PTEN expression compared with NC control (Figure 4B). A group of vehicle control (0.01 M PBS) was also used in the following study. In social paradigm for LTM-guided social recognition (Figure 4C1), we found that PTEN-shRNA, NC and vehicle groups were all exhibiting normal sociability (F *vs.* E) in trial 2 (Figures 4C2,C3, ^∗∗∗^*P* < 0.001) and intact social recognition when exploring F *vs.* S in trial 3 when the ITIs were set for 5- or 30-min (Figures 4C4,C5, ^∗∗^*P* < 0.01). In contrast, in social paradigm for STM-guided social recognition (Figure 4D1), PTEN-shRNA, NC and vehicle groups were all exhibiting normal sociability (S1 *vs.* E) in trial 2 (Figures 4D2,D3, ^∗∗∗^*P* < 0.001), but PTEN-shRNA group showed impaired social recognition (S1 *vs.* S2) in trial 3 when the ITI was set at either 1- or 5-min (Figures 4D4,D5, ^∗∗^*P* < 0.01, ^∗∗∗^*P* < 0.001). Normal social recognition showing preference to S2 over S1 was not observed in all groups when the ITI was set at 30 min (Figures 4D4,D5). But these deficits were not contributed by olfactory malfunction since all groups displayed normal in olfactory test (Figure 4F). The locomotor activity was not significantly different among groups (Supplementary Figure 7D). Notably, all groups displayed normal functions in non-social object recognition tasks (Supplementary Figures 2A,B). Furthermore, in T-maze spontaneous alternation test which is a spatial STM task sensitive to dorsal hippocampus dysfunction (Deacon et al., 2002; Deacon and Rawlins, 2006), PTEN-shRNA group exhibited lower performance scores (Figures 4E1,E2, ^∗^*P* < 0.05, ^∗∗^*P* < 0.01). We also employed a GO/NO GO task to test the non-spatial STM/WM using the technique described previously (Liu et al., 2014) in the CA1 PTEN CKO mouse model of ASD that was generated by injecting AAV-CaMKII-cre into bilateral dorsal CA1 regions of the *Pten*^*loxP/loxP*^ mice (Supplementary Figure 3A). STM-guided social recognition deficit was also found in these CA1 PTEN CKO mice (Supplementary Figures 3B1–B3), like those found in CA1 PTEN KD mice. The locomotor activity was not significantly different between groups (Supplementary Figure 7E). However, we did not find any change in a GO/NO GO task in CA1 PTEN CKO relative to WT mice (*Pten*^*loxP/loxP*^) (Supplementary Figure 4). These findings suggested that selective CA1 KD of PTEN led to deficits in social STM-guided social recognition.

### CaMKII-cre PTEN CKO in Social Recognition

**FIGURE 5:**
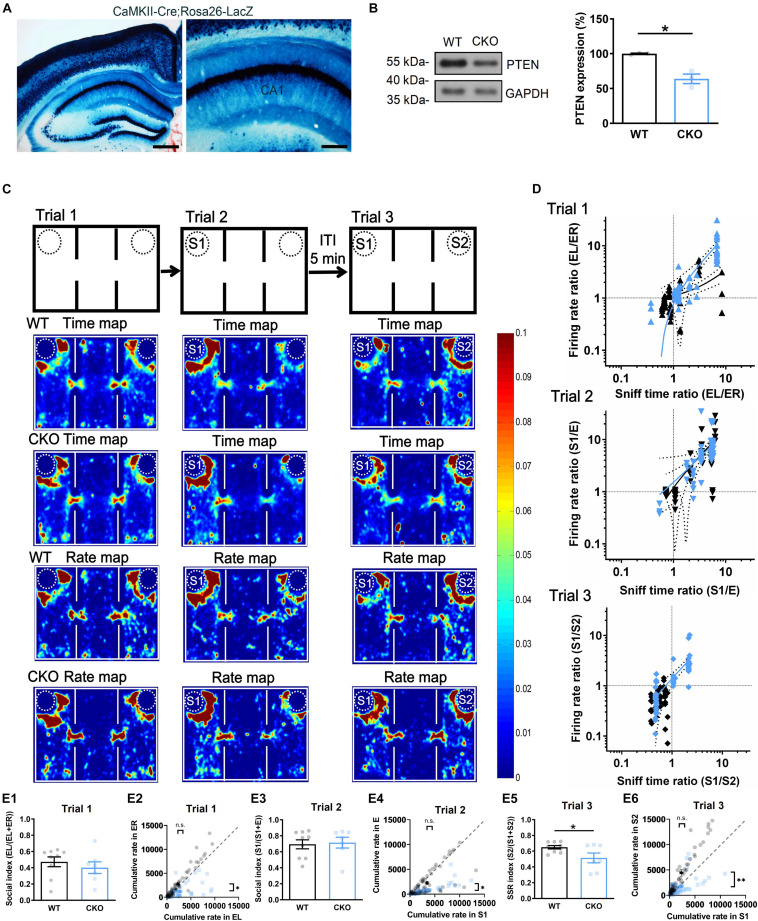
Altered CA1 neuronal firing pattern during social recognition in conditional forebrain PTEN knockout (CKO) mice. **(A)** X-Gal staining of Cre activity in CaMKII-Cre;Rosa26-LacZ mouse. Calibration bars: Left, 500 μm; Right, 200 μm. **(B)** Western blot of the dorsal CA1 samples from PTEN CKO mice showed down-regulation of PTEN expression relative to WT mice (*n* = 3/group; *P < 0.05). **(C)** Wild type (WT) and PTEN CKO mice were examined in three-chambers and three-trials social interaction test during which activity of CA1 neurons were recorded by using a multiple single-unit recording system and behavioral tracks of the mice were recorded by using a video system. Time map showing where subject mice spend their time; Rate map displaying where CA1 neuron is firing at a rate. Warmer colors represent more time or higher firing rate during 10-min per trial. Accumulated data (60 neurons from 9 WT; 59 neurons from 7 CKO) suggested a highly association between time map and rate map, but no difference between PTEN CKO and WT mice in trial 1 and 2. However, these maps showed a marked preference to S2 over S1 in WT mice but not in PTEN CKO mice in trial 3. **(D)** Empty cage in left side (EL)/empty cage in right side (ER) or a first stranger (S1)/empty cage **(E)** in the other side or a first stranger (S1)/a second stranger (S2) were used to calculate sniff time ratio or firing rate ratio, which were further plotted against each other. The distribution of the quantification dots in trial 1 and trial 2 was hardly separated for PTEN CKO (blue) from WT mice (black). Linear regression in Trial 2, *Y* = 1.645*X - 0.4547 for WT, *Y* = 1.393*X + 0.1245 for CKO, *P* = 0.6131 for slope comparison, *P* = 0.7253 for elevation comparison; Linear regression in Trial 3, *Y* = 0.7190*X + 0.1566 for WT, *Y* = 1.549*X - 0.1612 for CKO, *P* = 0.4197 for slope comparison, *P* = 0.4508 for elevation comparison. However, the quantification dots were clearly separated as two clusters in PTEN CKO that indicated no preference to S2 over S1, but those were kept in one cluster in WT mice that indicated preference to S2 over S1. **(E1)** Social index was similar between WT and PTEN CKO in trial 1 (*n* = 9 for WT, *n* = 7 for CKO, *t* = 0.7669, *P* = 0.4559). **(E2)** Cumulative rate was similar between WT EL and CKO EL in trial 1. Cumulative rate was higher in WT ER relative to CKO ER (*n* = 60 single units for WT, *n* = 59 single units for CKO; WT EL vs. CKO EL, *P* = 0.4191; WT ER vs. CKO ER, *P* = 0.0185). **(E3)** Social index was similar between WT and PTEN CKO in trial 2 (*n* = 9 for WT, *n* = 7 for CKO, Mann Whitney’s *U* test, *P* > 0.9999). **(E4)** Cumulative rate was similar between WT S1 and CKO S1 in trial 2. Cumulative rate was higher in WT E relative to CKO E (*n* = 60 single units for WT, *n* = 59 single units for CKO, WT S1 vs. CKO S1, *P* = 0.5675; WT E vs. CKO E, *P* = 0.0106). **(E5)** SSR index was significantly different between WT and PTEN CKO in trial 3 (*n* = 9 for WT, *n* = 7 for CKO, *t* = 2.258, *P* = 0.0405). **(E6)** Cumulative rate was similar between WT S1 and CKO S1 in trial 3. Cumulative rate was higher in WT S2 relative to CKO S2 (*n* = 60 single units for WT, *n* = 59 single units for CKO, WT S1 vs. CKO S1, *P* = 0.7018; WT S2 vs. CKO S2, *P* = 0.003; **P < 0.01, *P < 0.05). EL, empty cage left; ER, empty cage right; E, empty cage; S1, the first stranger mouse; S2, the second stranger mouse. Data presented as mean ± SEM. Statistical analysis was performed by using Mann Whitney’s *U* test, Kolmogorov–Smirnov test and student’s *t* test.

To verify whether dorsal CA1 neuronal activity is disrupted *in vivo* during the behavioral study, we further used CaMKII-cre mice and *Pten*^*loxP/loxP*^ mice to generate the mice with *Pten* CKO in the forebrain and the hippocampus (Figure 5A) starting at postnatal 2 weeks similar as the PTEN mice reported previously (Sperow et al., 2012; Garcia-Junco-Clemente et al., 2013). Western blot revealed about 36.1 ± 7.1% reduction of PTEN expression in PTEN CKO relative to WT mice, in the CA1 region of the dorsal hippocampus (Figure 5B). Then, CKO and WT mice were examined in the social behavior study, and the mice with the *Pten^+/+^* or *Pten^+/loxP^* genotypes were used as the stranger (S) mouse. We directly recorded dorsal CA1 neuronal activity through a multi-electrode array system and videoed behavioral trajectory in freely-moving CKO and WT mice during the behavioral test (Supplementary Figure 5A). Spike sorting and analysis were carried out off-line (Supplementary Figures 5B,C). According to behavioral trajectory, a time map was plotted for indicating where the subject mice spend their time during the trials. According to CA1 neuronal firing, a rate map was plotted for showing where CA1 neurons fire at a rate (Figure 5C). The warmer colors indicated as more exploration time or higher neuronal firing rate. All data from each animal were accumulated together (60 neurons from 9 WT; 59 neurons from 7 CKO). Notably, CKO and WT mice exhibited very much similar patterns in both the maps in trial 1 and trial 2, suggesting that rate map for CA1 neuronal activity may actually reflect time map for exploration of the subject mice, which may be consistent with the concept that “a cognitive map” could have guided behavior (O’Keefe and Nadel, 1978; Manns and Eichenbaum, 2009). In contrast, the patterns of the maps were largely different between CKO and WT mice during trial 3 (Figure 5C, trial 3), indicating that impaired social recognition in CKO mice was associated with their altered CA1 neuronal firing pattern. A recent study indicates that CA1 neuronal firing can predict the direction of behaving mice where to move in the next steps (Pfeiffer and Foster, 2013). Accordingly, we believed that the altered firing pattern of dorsal CA1 neurons in behaving CKO mice was likely responsible for the altered social recognition in trial 3. In Figure 5D, two orthogonal lines starting from *X* = 1 (Sniff time ratio) and *Y* = 1 (Firing rate ratio) divided the plane into four sub-quadrants. In trial 1 of Figure 5D, the black dots (WT) and blue dots (CKO) were mostly overlaid near the intersection and founded in all four sub-quadrants, indicating similar firing rate ratio-sniff time ratio correlation between WT and CKO. In trial 2, most of the black dots (WT) were above 1 (S1/E > 1), suggesting higher firing rate around S1 relative to E. The trend was similar in blue dots (CKO), suggesting similar firing rate- sniff time correlation between WT and CKO. However, in trial 3, most of the black dots (WT) were below 1 indicating lower firing rate around S1 relative to S2 while some blue dots (CKO) were higher than 1 indicating higher firing rate around S1 relative to S2. Thus, while the correlation showed that the higher firing rate, the higher sniff time in both WT and CKO mice (Linear regression in Trial 2, *Y* = 1.645^∗^X - 0.4547 for WT, *Y* = 1.393^∗^X + 0.1245 for CKO, *P* = 0.6131 for slope comparison, *P* = 0.7253 for elevation comparison; Linear regression in Trial 3, *Y* = 0.7190^∗^X + 0.1566 for WT, *Y* = 1.549^∗^X - 0.1612 for CKO, *P* = 0.4197 for slope comparison, *P* = 0.4508 for elevation comparison), CKO mice showed altered firing pattern regarding higher firing rate towards S1 compared to that in WT mice. Consistent with this result, further analysis found that behavioral social index were nearly identical in trial 1 (Figure 5E1) and trial 2 (Figure 5E3), but significantly lower in *Pten*^–/–^ mice compared with that in WT mice in trial 3 (Figure 5E5, ^∗^*P* < 0.05). Also, firing rate towards two empty cages in trial 1 was similar in WT neurons. But neurons in *Pten*^–/–^ mice exhibited lower rate towards ER (Figure 5E2). While firing rate towards S1 in trial 2 was similar between two groups (Figure 5E4), neurons in *Pten*^–/–^ mice exhibited lower rate towards S2 in trial 3 (Figure 5E6, ^∗∗^*P* < 0.01). The locomotor activity was not significantly different between groups (Supplementary Figure 7F). In addition, both CKO and WT mice showed normal social recognition with the ITI for 5- or 30-min by referring to F (F *vs.* E in trial 2 and F *vs.* S in trial 3) (Supplementary Figure 6A), and normal capability in non-social object recognition tasks (Supplementary Figures 6B,C) and olfactory test (Supplementary Figure 6D), similar to the above findings in PTEN KD mice (Supplementary Figure 2). Thus, we demonstrated that dorsal CA1 neurons showed a lower firing rate *in vitro* and altered firing pattern *in vivo* during social recognition test in PTEN KD or CKO mice.

**FIGURE 6:**
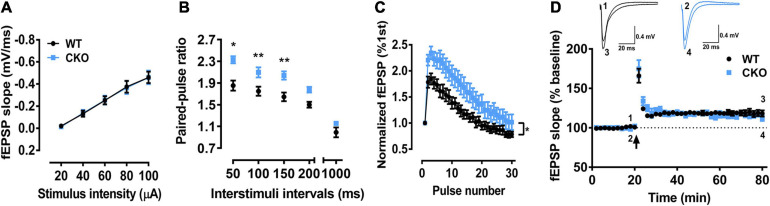
The field excitatory postsynaptic potentials (fEPSP) recordings in CA1 region of PTEN CKO mice in brain slices. **(A)** The input-output curve in CA1 by stimulating Schaffer-collaterals was not significant difference between PTEN CKO and WT mice (*n* = 12 from 5 mice for WT, *n* = 18 from 7 mice for CKO; Main effect of stimuli, *F*_(4,112)_ = 85.57, *P* < 0.0001; Main effect of group, *F*_(1,28)_ = 0.00065, *P* = 0.9798; Interaction effect of group x stimuli, *F*_(4,112)_ = 0.02257, *p* = 0.9990). **(B)** Paired-pulse facilitation was significantly enhanced in PTEN CKO mice as compared with WT mice (*n* = 6 from 3 mice for WT, *n* = 7 from 3 mice for CKO; Main effect of interval, *F*_(4,44)_ = 98.89, *P* < 0.0001; Main effect of group, *F*_(1,11)_ = 18.93, *P* = 0.0012; Interaction effect of group x interval, *F*_(4,44)_ = 2.241, *P* = 0.0799. Sidak’s *post hoc* test. *P* = 0.0159 at 50 ms; *P* = 0.0074 at 100 ms; *P* = 0.0019 at 150 ms; *P* = 0.0507 at 200 ms; *P* = 0.4654 at 1000 ms). **(C)** Short-term augmentation at 20 Hz was also significantly enhanced in PTEN CKO mice as compared with WT mice (*n* = 9 from 3 mice for WT, *n* = 11 from 4 mice for CKO; Main effect of stimuli, *F*_(29,522)_ = 88.3, *P* < 0.0001; Main effect of group, *F*_(1,18)_ = 4.576, *P* = 0.0464; Interaction effect of group x stimuli, *F*_(29,522)_ = 2.524, *P* < 0.001). **(D)** Long-term potentiation was not significantly different between PTEN CKO and WT mice (*n* = 13 from 6 mice for WT, *n* = 8 from 4 mice for CKO; Last 2 min, *t* = 1.043, *P* = 0.3096; *P* > 0.05). Data presented as mean ± SEM. Statistical analysis was performed by using student’s *t* test or Two-way ANOVA followed by Sidak’s *post hoc* analysis.

Since partial dorsal CA1 neurons are PTEN CKO (Figure 5B), we recorded the fEPSP only in brain slices. We found that input-output curve for testing basal synaptic weight remained unchanged (Figure 6A), but two forms of short-term synaptic plasticity, paired-pulse facilitation (Figure 6B, ^∗^*P* < 0.05, ^∗∗^*P* < 0.01) and short-term augmentation at 20 Hz (Figure 6C, ^∗^*P* < 0.05) were both pathologically enhanced, suggesting the function of presynaptic CA3 Schaffer-collateral projections was affected by PTEN deletion. In addition, LTP induction for testing long-term synaptic plasticity (Figure 6D) also remained unchanged in PTEN CKO mice, which was similar as that found in PTEN KD mice (see Figure 3I).

Altogether, our present study led to the conclusion that a temporal activity of dorsal CA1 neurons may be a critical mechanism underlying STM to organize social recognition under 30 min in WT mice but that is likely disrupted in PTEN mouse models of ASD.

## Discussion

In the present study, we found that STM is likely represented by a temporal activity of dorsal CA1 neurons under 30 min to be critical for subsequent social recognition, but that was possibly disrupted in three PTEN mouse models of ASD (PTEN KD and PTEN CKO in the dorsal hippocampal CA1 regions, PTEN CKO in the cortex and the hippocampus). In our previous study, we found that P-REX1 mouse model of ASD exhibit disrupted CA1 synaptic plasticity and ASD-like social recognition (Li et al., 2015). Therefore, ASD-like social recognition can be originated from distinct associated genes (Geschwind and State, 2015), attributable to disruption of either a temporal activity of CA1 neurons or CA1 synaptic plasticity.

### WM, STM, and LTM for Social Recognition

Social interaction test with the three-chamber and three-trial is widely used for testing social behaviors in mice, especially the sociability test in trial 2 and social recognition in trial 3. Here, we found that STM was likely formed by exploring S1 and related context in trial 2 so that subject mice become familiar to S1 (Figure 1B). Thus, STM holds the information for minutes because the interval at 1 or 5 min but not 30 min or 6 h can endow normal social recognition in trial 3 by exploring S1 *vs.* S2 and exhibiting preference to S2 over S1 (Figure 1B). However, because subject mice have formed LTM about F, this limitation for the interval by 1 or 5 min can be prolonged to 30 min or 6 h, suggesting that LTM can also guide social recognition (Figure 1A). Furthermore, WM is believed able to manipulate STM or LTM so that social recognition is likely to also engage WM (Brown et al., 2008; Meyer et al., 2012). It is notable that dorsal CA1 PTEN KD mice exhibited impaired social recognition and WM in T-maze (Figure 4E). Thus, we believe that any one of WM and STM and LTM is critical for social recognition, but here disrupted STM (Figure 4D and Supplementary Figures 3B3, 5E) but not LTM (Figure 4C and Supplementary Figure 6A) is responsible for ASD-like social recognition in PTEN mouse models of ASD.

### Social Recognition and Hippocampus

Social memory is the formation, storage and expression of social information. It has been reported that rodents acquire social information through various sensory cues, including olfactory (Oettl et al., 2016), auditory (Rao et al., 2014), and tactile (Lenschow and Brecht, 2015) so as to establish the identity of individuals. Dorsal hippocampus receives polymodal sensory information from cortical areas and plays an important role in contextual/spatial information process (Kim and Fanselow, 1992; Moser et al., 1993; Fanselow and Dong, 2010). We found optogenetic inhibition of dorsal CA1 during trial 2 impaired social recognition (Figure 2D). Why? Bilateral injection of protein synthesis inhibitor or monoamine antagonists in dorsal CA1 after training led to social recognition deficit 24 h later in Wistar rats (Garrido Zinn et al., 2016). While it has been reported that most dorsal CA1 cells were weakly modulated by conspecific presence (von Heimendahl et al., 2012), significant learning-induced immediate-early gene c-fos and Acr were observed in dorsal CA1 when STM/LTM for social recognition was generated (Tanimizu et al., 2017). Besides, recent studies provided evidence that dorsal CA1 place cells encoded the spatial location of conspecifics (Danjo et al., 2018; Omer et al., 2018). Our findings showed that optogenetic inhibition of dorsal CA1 during trial 2 did not affect sociability. Hence, the impaired social recognition was due not to diminished sociability, but possibly to disturbed social information processing, e.g., social information coordination. There are also emerging evidence supporting a “CA2-ventral CA1” brain circuit in which long-term social memory was regulated. Chemogenetic inhibition of CA2 activity impaired long-term social memory with littermate and direct social interaction-induced social memory which could last over 24 h (Kogan et al., 2000; Hitti and Siegelbaum, 2014). Follow-up work with chemogenetic and optogenetic silencing of CA2-ventral CA1 activity resulted in impaired encoding, consolidation and expression of long-term social memory (Meira et al., 2018). Another study has shown that optogenetic manipulation of mouse ventral, but not dorsal hippocampal CA1 was necessary and sufficient for expression of a direct social interaction-induced social memory lasting more than 30 min (Okuyama et al., 2016). Another study with optogenetic silencing of CA2-ventral CA1, not CA2-dorsal CA1 resulted in social discrimination impairment in a social habituation-dishabituation behavioral paradigm (Raam et al., 2017), the longevity of this social memory was not clear. These findings indicated that CA2-ventral CA1 was crucial for social LTM last more than 30 min. We also found that social recognition expression was intact with social position change (Supplementary Figures 1C,D). Then, we used optogenetic tool to explore whether STM is dependent on a temporal activity of CA1 neurons that is critical for social recognition in trial 3. We found that optogenetic silencing of CA1 neuronal activity either during the ITI before trial 3 (Figure 2C) or during trial 2 (Figure 2D) similarly led to impaired STM-guided social recognition in trial 3, while sociability and LTM-guided social recognition were intact (Figure 2E). We found that social STM impairment in PTEN KD (Figure 4) and CKO mice (Figure 5), probably due to decreased dorsal CA1 neuronal firing (Figure 3) or altered dorsal CA1 neuronal firing pattern, respectively (Figure 5). These novel finding provides strong evidence for which a temporal activity of dorsal CA1 neurons is critical for STM formation to guide following social recognition. It has been reported that dorsal CA1 receives direct synaptic projection from CA2, and functionally connects with ventral CA1 through indirect projection from nucleus reuniens (Cui et al., 2013; Varela et al., 2014), thus it would be interesting to test the role of dorsal CA1-CA2 involved in formation of social STM in further investigation.

It should be noted that current results in dorsal hippocampal CA1 do not rule out the critical roles of other brain regions in forebrain in regulating social recognition. For instance, some studies have found other forebrain regions, including olfactory bulb, medial prefrontal cortex (mPFC), amygdala and hypothalamus are activated after social recognition. Pharmacological, optogenetic and/or genetic manipulations of neurons in these regions could modulate social recognition in the animals (Ferguson et al., 2001; Tobin et al., 2010; Jacobs and Tsien, 2014; Cao et al., 2018a; Niu et al., 2018). Therefore, further efforts are still needed in dissecting the role of PTEN in these specific forebrain regions during social interaction.

It is not clear yet why STM for social recognition in mice is limited by a time period of less than 30 min. One possibility is that limited modalities in three-chamber behavioral test may restrict the retention of STM (TindallFord et al., 1997), while direct social interaction-induced social memory could last at least 24 h (Kogan et al., 2000; Meira et al., 2018). Another possibility is that the information is rapidly updating from trial 1 to 3. This may cause proactive and retroactive interference to STM that may restrict the retention of STM. We reasoned that the impairment could be also considered as interference to STM about S1. It is possible that STM holds information only temporally so that it is easy to have interfered.

### PTEN-Dependent Neural Plasticity and Social Recognition

We used three PTEN mouse models of ASD because mutations of *PTEN* are associated with ASD, and PTEN CKO in mice can replicate many aspects of ASD (Backman et al., 2001; Kwon et al., 2001; Fraser et al., 2008). Sociability in trial 2 is impaired in PTEN CKO mice as reported by those (Kwon et al., 2006; Amiri et al., 2012) but not in others (Page et al., 2009), possibly due to different experimental conditions since Nestin-Cre, GFAP-Cre, Nse-Cre targeted different neuronal populations and activated at different developmental stages, since Cre expression in the CamKIIα-Cre line starts at postnatal 2 weeks. Nevertheless, these studies have demonstrated a highly consistent deficit of social recognition in trial 3. Here, we used PTEN mouse models of ASD and found highly consistent results for which sociability in trial 2 remained unchanged but social recognition in trial 3 was impaired (Figures 4, 5). In contrast, PTEN and WT mice are not different in object recognition tasks and olfaction test (Supplementary Figures 2, 6), consistent with a previous report (Kwon et al., 2006). It is notable that dorsal CA1 PTEN CKO mice also exhibited the normal function of WM in a non-social GO/NO GO task for testing WM (Supplementary Figure 4).

The underlying mechanisms for ASD-like social recognition in PTEN mice may attribute to abnormalities of cell morphology (Haws et al., 2014), cell migration (Marino et al., 2002), neurogenesis (Groszer et al., 2001; Amiri et al., 2012), and synaptic plasticity (Wang et al., 2006; Fraser et al., 2008; Jurado et al., 2010). Furthermore, the developmental deficit is important for ASD, although it may occur after functional changes (Kwon et al., 2006). Since CaMKII-Cre PTEN CKO occurs substantially after postnatal development (Tsien et al., 1996a), it suggests that ASD-like social recognition is unlikely attributable to the developmental abnormalities only. Moreover, deficits of synaptic functions might also precede the anatomical changes (Luikart et al., 2011; Sperow et al., 2012; Takeuchi et al., 2013). Synaptic plasticity and neuronal excitability were two forms of neural plasticity and served as cellular mechanisms of learning and memory (Daoudal and Debanne, 2003). Short-term synaptic plasticity has been postulated as a cellular mechanism for STM and/or WM (Brunelli et al., 1976; Zucker and Regehr, 2002; Abbott and Regehr, 2004; Mongillo et al., 2008; Hansel and Mato, 2013). In this study, as for short-term plasticity which mainly reflected presynaptic activity (Zucker and Regehr, 2002), we found short-term facilitation and augmentation were intact in PTEN CA1 KD slices, but pathologically enhanced in forebrain CKO slices (Supplementary Figure 6), indicating forebrain PTEN CKO affected presynaptic CA3 activity. However, other studies found short-term plasticity was either intact, or impaired, or enhanced in hippocampus. One possibility of this discrepancy could be due to animal lines and animal ages since synaptic plasticity changed along with development (Wang et al., 2006; Sperow et al., 2012; Takeuchi et al., 2013). Furthermore, we found long-term potentiation was intact in PTEN KD and CKO which was in line with the results from PTEN inhibitor-treated slices and PTEN heterozygous mice (Wang et al., 2006; Jurado et al., 2010), but not with others (Fraser et al., 2008; Sperow et al., 2012). Different LTP induction protocols may account for this discrepancy since 200 Hz could induce NMDA-receptor-independent potentiation (Grover and Teyler, 1990; Cavus and Teyler, 1996). As for neuronal excitability, some evidences support another notion that intrinsic neuronal excitability may underlie STM (Fuster and Alexander, 1971; Marder et al., 1996). We found reduced firing rate *in vitro* with CA1 PTEN KD (Figure 3) which was consistent with the findings of reduced firing rate in PTEN haploinsufficient L2/3 pyramidal neurons and in PTEN CKO purkinje neurons (Garcia-Junco-Clemente et al., 2013; Cupolillo et al., 2015), but not with the finding of enhanced firing rate in PTEN CKO dentate gyrus granule cells (Williams et al., 2015). We also found AHP enhanced in CA1 PTEN KD neurons (Figure 3). How did diminished PTEN expression lead to lower neuronal excitability with regard to less action potential firings and larger AHP? PTEN acts as a suppressor of the PI3K/AKT/mTOR signaling pathway which involves in regulating transcription factors. Thus, one possibility is that neuronal excitability decreased due to PI3K/AKT/mTOR signaling hyperactivity after PTEN reduction. A recent study supported this hypothesis by showing that PTEN loss lead to increased level of AKT/mTOR signaling and Kv4.2 which may lower the excitability (Carrasquillo et al., 2012; Nolan et al., 2019). Furthermore, AHP could be mediated by calcium-activated potassium channels (e.g., small conductance calcium-activated potassium channel, SK channel) (Sah, 1996; Bean, 2007). It has been reported that neural excitability could be regulated through direct binding between SK channel and FMRP, and the expression level of both proteins in PTEN deleted neurons increased several folds (Garcia-Junco-Clemente et al., 2013; Deng et al., 2019; Nolan et al., 2019). Therefore, another possibility is that PTEN loss lead to larger AHP by increasing the activity and/or expression level of SK channel. Further studies will be required to address whether modulation of these molecular mechanisms and channels could rescue neuronal excitability and social recognition deficits in PTEN CKO mice.

It is interesting that our previous report showed that KO of *P-REX1*, an ASD associated gene newly found in Chinese Han children, leads to impaired CA1 synaptic plasticity long-term depression (LTD) and impaired social recognition, without any alteration in firing rate of action potentials in CA1 *in vitro* (Li et al., 2015). Using similar methods, we examined PTEN mice. PTEN mice exhibited reduced firing rate in CA1 *in vitro* or altered firing pattern *in vivo* during social recognition task. Because our optogenetic study demonstrated that a temporal activity of CA1 neurons during trial 2 or during the interval before trial 3 was critical for social recognition (Figure 2), we suggest that a temporal activity of CA1 neurons could be the mechanism underlying STM formation for social recognition. How did two different cellular mechanisms, long-term depression and neuronal excitability, contribute to same behavioral phenotype such as STM-guided social recognition? It has been shown that persistent firing was related to information holding during delay in STM/WM (Fuster and Alexander, 1971; Liu et al., 2014). In addition to our previous finding, synaptic depression in mushroom body or long-term depression in perirhinal cortex was underlying expression of familiarity-dependent behaviors, such as novelty alerting and visual recognition (Griffiths et al., 2008; Hattori et al., 2017). A recent human research revealed that unattended short-term information could be held via “activity-silent” synaptic mechanism before further WM manipulation, while attended information was still held by sustained, elevated neural activity (Rose et al., 2016). Thus, it is possible that during three-chamber task, social information were held temporally via different neural mechanisms, including neuronal excitability and synaptic depression, and was impaired in PTEN or P-Rex1 mouse model of ASD with least one of them.

In summary, we demonstrate strong novel evidence suggesting that a temporal activity of CA1 neurons underlies STM formation to be critical for social recognition. In contrast, this mechanism for STM formation is possibly disrupted in three PTEN mouse models of ASD by showing CA1 neurons with lower firing rate *in vitro* and altered firing pattern during social recognition test. Nevertheless, altered CA1 neuronal activity may further influence neuronal activity in neural circuits among the interconnected brain regions with the hippocampus (Huang et al., 2016). This could be a key reason why mouse models of ASD share a common behavioral phenotype despite distinct pathophysiological mechanisms that originated from different ASD associated genes.

## Data Availability Statement

The raw data supporting the conclusions of this article will be made available by the authors, without undue reservation.

## Ethics Statement

The animal study was reviewed and approved by Institutional Animal Care and Use Committee of Kunming Institute of Zoology, the Chinese Academy of Sciences.

## Author Contributions

A-PC was responsible for the initial findings, performed most of the experiments, and wrote the draft. X-FC performed MEA recordings and analyses. X-SX performed the GO/NO GO task for testing WM. NZ, ML, J-NL, and LZ assisted IHC and electrophysiological studies. DZ, XZ, R-RM, and Y-QD contributed to critical discussions and manuscript editing. LX and Q-XZ designed and directed the research and wrote the manuscript, which was reviewed by all authors.

## Conflict of Interest

The authors declare that the research was conducted in the absence of any commercial or financial relationships that could be construed as a potential conflict of interest.
