# Supplementary material for: A Temporal Activity of CA1 Neurons Underlying Short-Term Memory for Social Recognition Altered in PTEN Mouse Models of Autism Spectrum Disorder
Source: Front Cell Neurosci. 2021 Jul 15;15:699315. doi: 10.3389/fncel.2021.699315 (PMC8319669; doi:10.3389/fncel.2021.699315)
Supplement: Supplementary file 1 [file Table_1.DOCX]

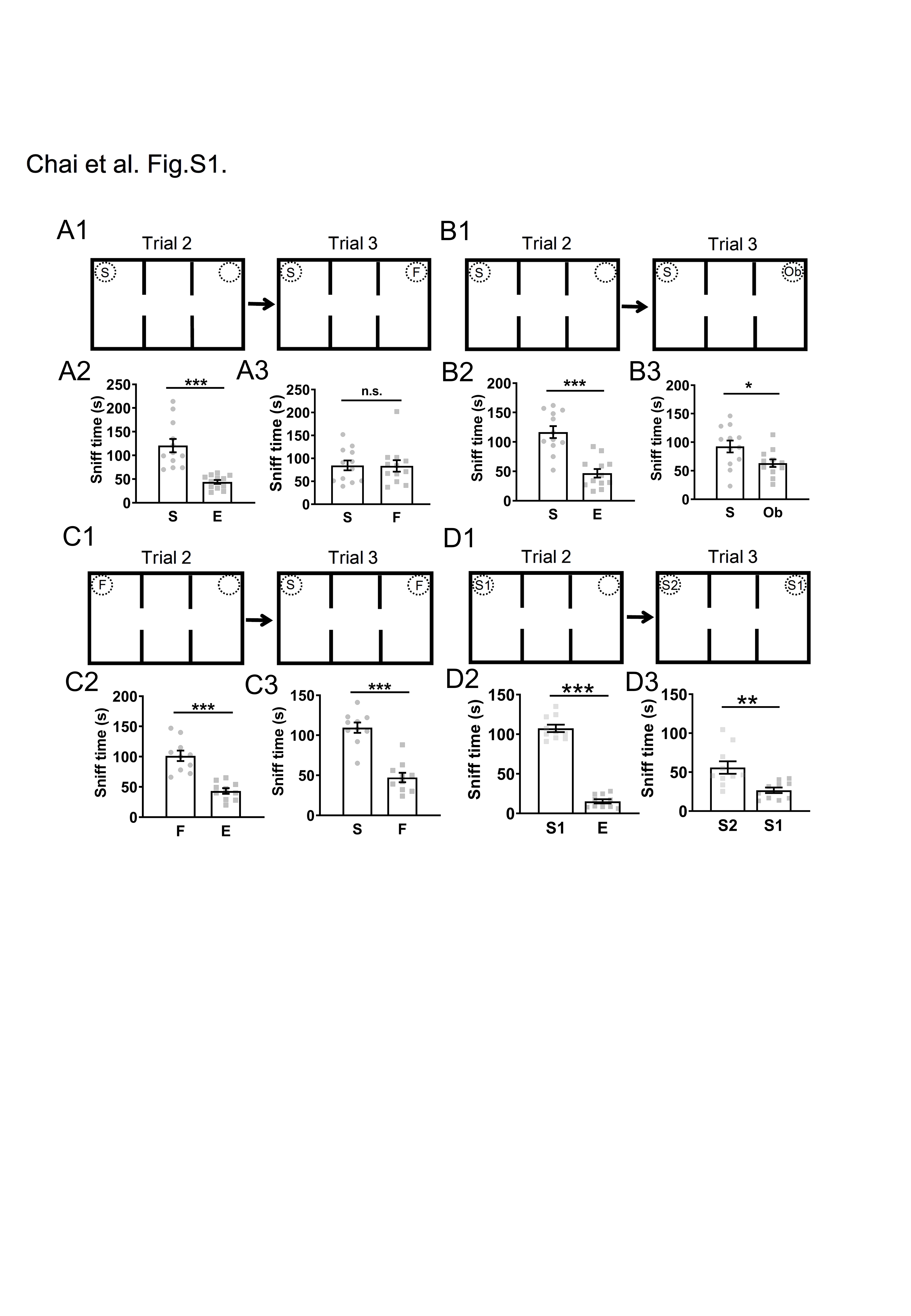


**Supplementary Figure 1. Short-term memory contributed to social recognition in mice.** (A1) Behavioral paradigm. (A2) Normal sociability (n = 12; t = 5.520, *P* < 0.0001 in trial 2). (A3) Social recognition in trial 3 by exploring a stranger mouse (S) *vs*. a familiar mouse (F) was impaired (n = 12; Wilcoxon test, *P* = 0.9863 in trial 3), suggesting occupation of formerly empty cage was insufficient to drive preference in social recognition. (B1) Social paradigm. (B2) Normal sociability in trial 2 (n = 12; t = 5.308, *P* < 0.0001 in trial 2). (B3) Social preference in trial 3 was even higher for S *vs.* a non-social novel object (Ob) (n = 12; t = 2.633, *P* = 0.0233 in trial 3). (C1) Social paradigm. (C2) Normal sociability with F (n = 9; t = 5.639, *P* < 0.0001 in trial 2). (C3) Long-term F memory-guided social recognition in trial 3 by exploring with S *vs.* F did not affect by location change of F (n = 9; t = 8.174, *P* < 0.0001). (D1) Social paradigm. (D2) Normal sociability (n = 10; t = 16.59, *P* < 0.0001). (D3) Short-term S1 memory-guided social recognition in trial 3 by exploring S2 *vs.* S1 was not affected by position change of S1 (n = 10; t = 3.738, *P* = 0.0046 in trial 3). **P* < 0.05; ***P* < 0.01; ****P* < 0.001; n.s., not significant. Data presented as mean ± SEM. Statistical analysis was performed by using Wilcoxon test and student’s *t* test.
