# Supplementary material for: A Temporal Activity of CA1 Neurons Underlying Short-Term Memory for Social Recognition Altered in PTEN Mouse Models of Autism Spectrum Disorder
Source: Front Cell Neurosci. 2021 Jul 15;15:699315. doi: 10.3389/fncel.2021.699315 (PMC8319669; doi:10.3389/fncel.2021.699315)
Supplement: Supplementary file 2 [file Table_2.DOCX]

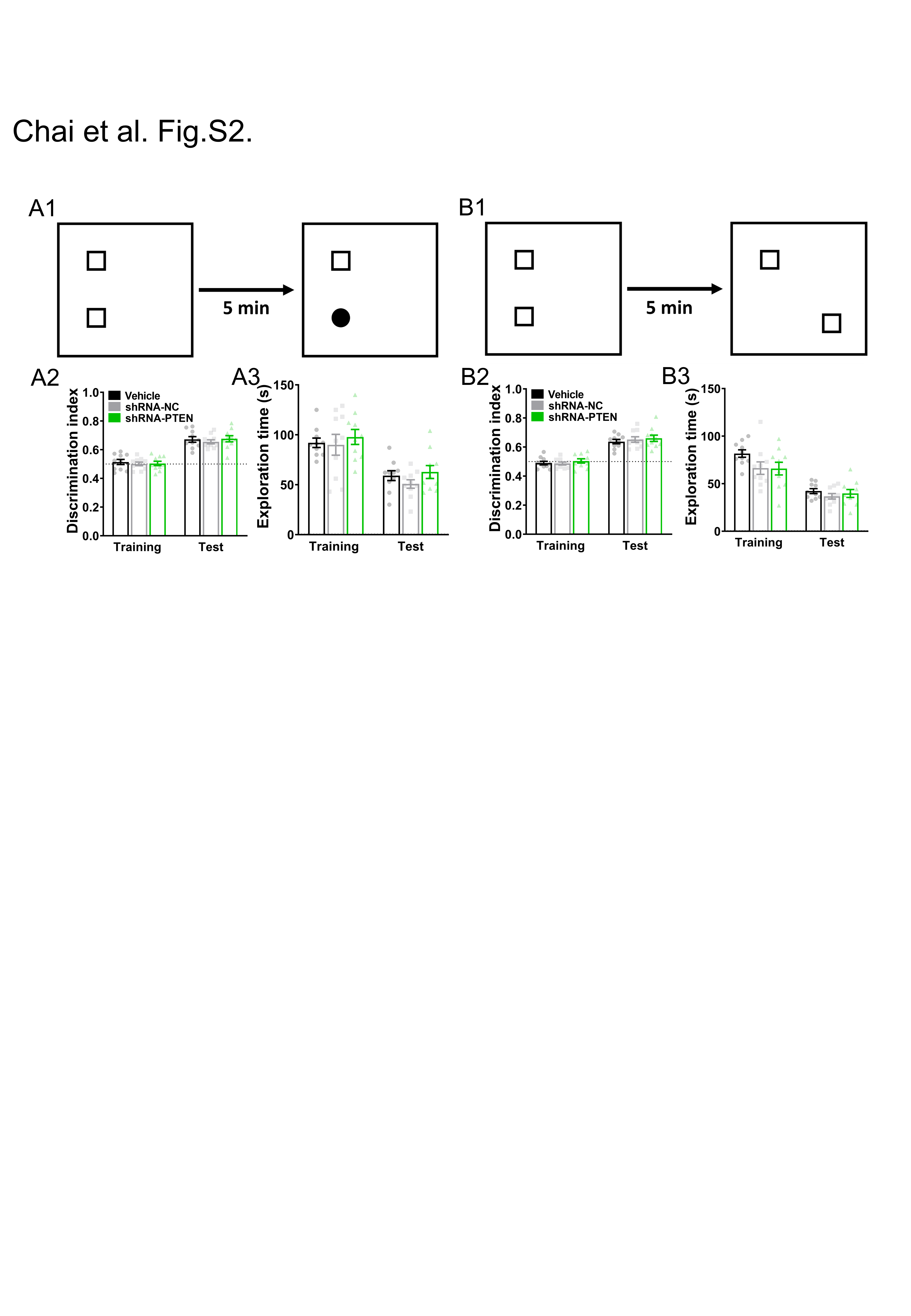


**Supplementary Figure 2. Object recognition in CA1 PTEN knock down (KD) mice.** (A) Subject mice with PTEN KD in the CA1 regions of the dorsal hippocampus exhibited normal shape-dependent object recognition compared to NC and Vehicle controls (n = 10/group. A2, Index training, *F*_(2,27)_=0.2022, *P* = 0.8182; Index test, *F*_(2,27)_ = 0.3634, *P* = 0.6987; A3, Exploration training, *F*_(2,27)_ = 0.2629, *P* = 0.7707; Exploration test, *F*_(2,27)_ = 1.348, *P* = 0.2767; *P* > 0.05). (B) CA1 PTEN KD mice showed normal position-dependent object recognition relative to NC and Vehicle controls (n = 10/group. B2, Index training, *F*_(2,27)_=0.5747, *P* = 0.5696; Index test, *F*_(2,27)_ = 0.3813, *P* = 0.6866; B3, Exploration training, Friedman test, *P* = 0.0665, χ2 = 5.600. Dunn's multiple comparisons test, Veh train vs. NC train, *P* = 0.2209; Veh train vs. PTEN train, *P* = 0.0760; NC train vs. PTEN train, *P* > 0.9999.). Data presented as mean ± SEM. Statistical analysis was performed by using one-way ANOVA followed by Tukey’s post hoc analysis, Friedman test followed by Dunn’s post hoc analysis.

.
