# Supplementary material for: A Temporal Activity of CA1 Neurons Underlying Short-Term Memory for Social Recognition Altered in PTEN Mouse Models of Autism Spectrum Disorder
Source: Front Cell Neurosci. 2021 Jul 15;15:699315. doi: 10.3389/fncel.2021.699315 (PMC8319669; doi:10.3389/fncel.2021.699315)
Supplement: Supplementary file 3 [file Table_3.DOCX]

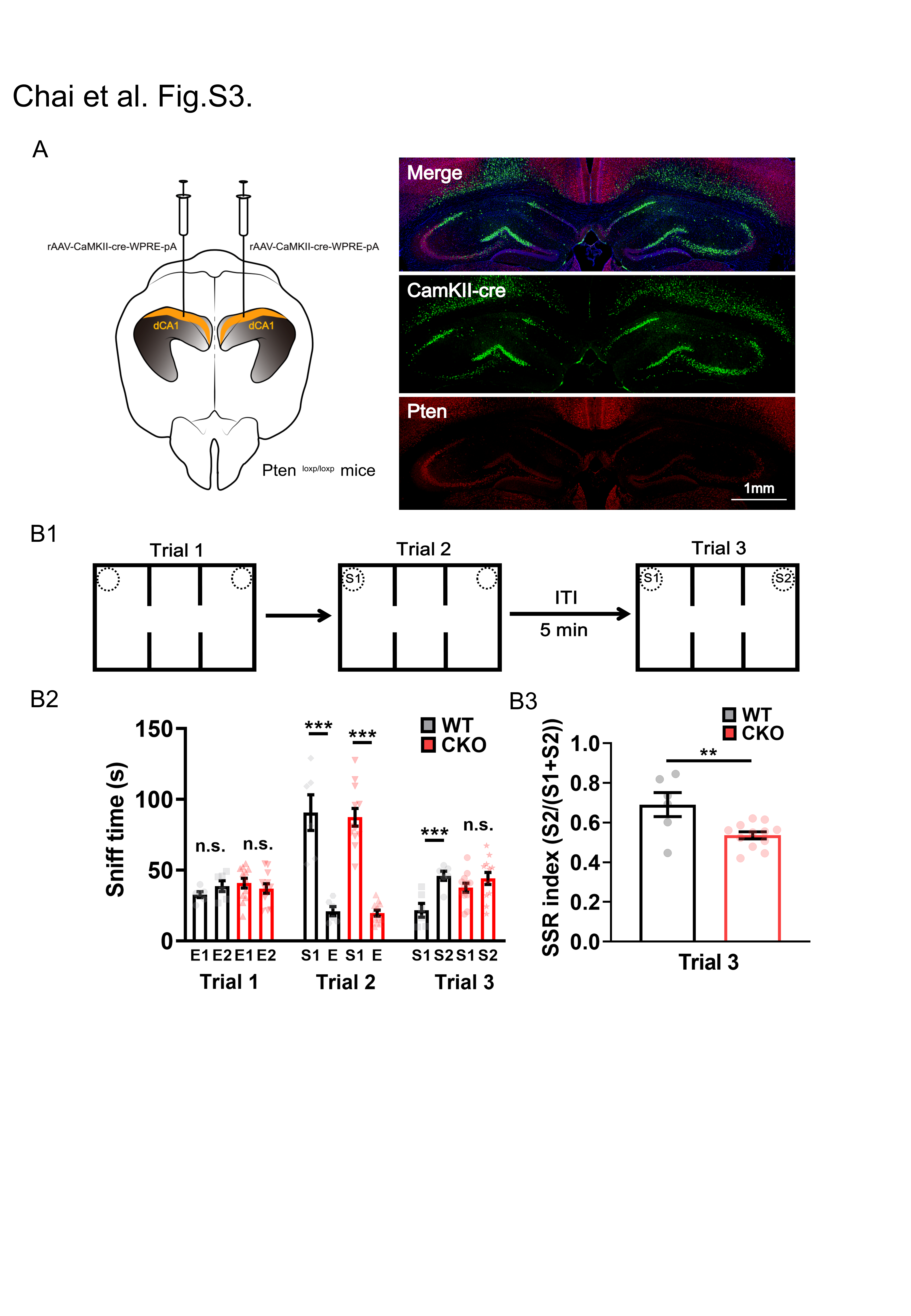


**Supplementary Figure 3. Hippocampal dorsal CA1 PETN CKO mice showed impaired social recognition.** (A) Left, the virus expressing Cre (rAAV-CaMKII-cre-WPRE-pA) was injected into the CA1 regions of the hippocampus for at least three weeks before experiments. Right, immunostaining indicated that PTEN expression was severely decreased in the dorsal CA1 regions, suggesting CA1 PTEN CKO. (B1) Experimental paradigm for the three-chamber and three-trial social interaction test. (B2) CKO and WT mice performed trial 1 by exploring first empty (E1) cage vs. second empty (E2) cage and trail 2 by exploring a first stranger mouse (S1) vs. an empty (E2) cage similarly well (n = 12 for CKO, n = 6 for WT; ****P* < 0.001 for both CKO and WT mice), but social recognition in trial 3 by exploring S1 vs. a second stranger mouse (S2) was impaired in CKO but not in WT mice (**P* = 0.0206 for WT; *P* > 0.05 for CKO). (B3) SSR index also indicated impaired social recognition in CKO but not in WT (CKO vs. WT, t = 3.159, *P* = 0.0061; ***P* < 0.01); n.s., not significant. Data presented as mean ± SEM. Statistical analysis was performed by using student’s *t* test.
