# Supplementary material for: A Temporal Activity of CA1 Neurons Underlying Short-Term Memory for Social Recognition Altered in PTEN Mouse Models of Autism Spectrum Disorder
Source: Front Cell Neurosci. 2021 Jul 15;15:699315. doi: 10.3389/fncel.2021.699315 (PMC8319669; doi:10.3389/fncel.2021.699315)
Supplement: Supplementary file 4 [file Table_4.DOCX]

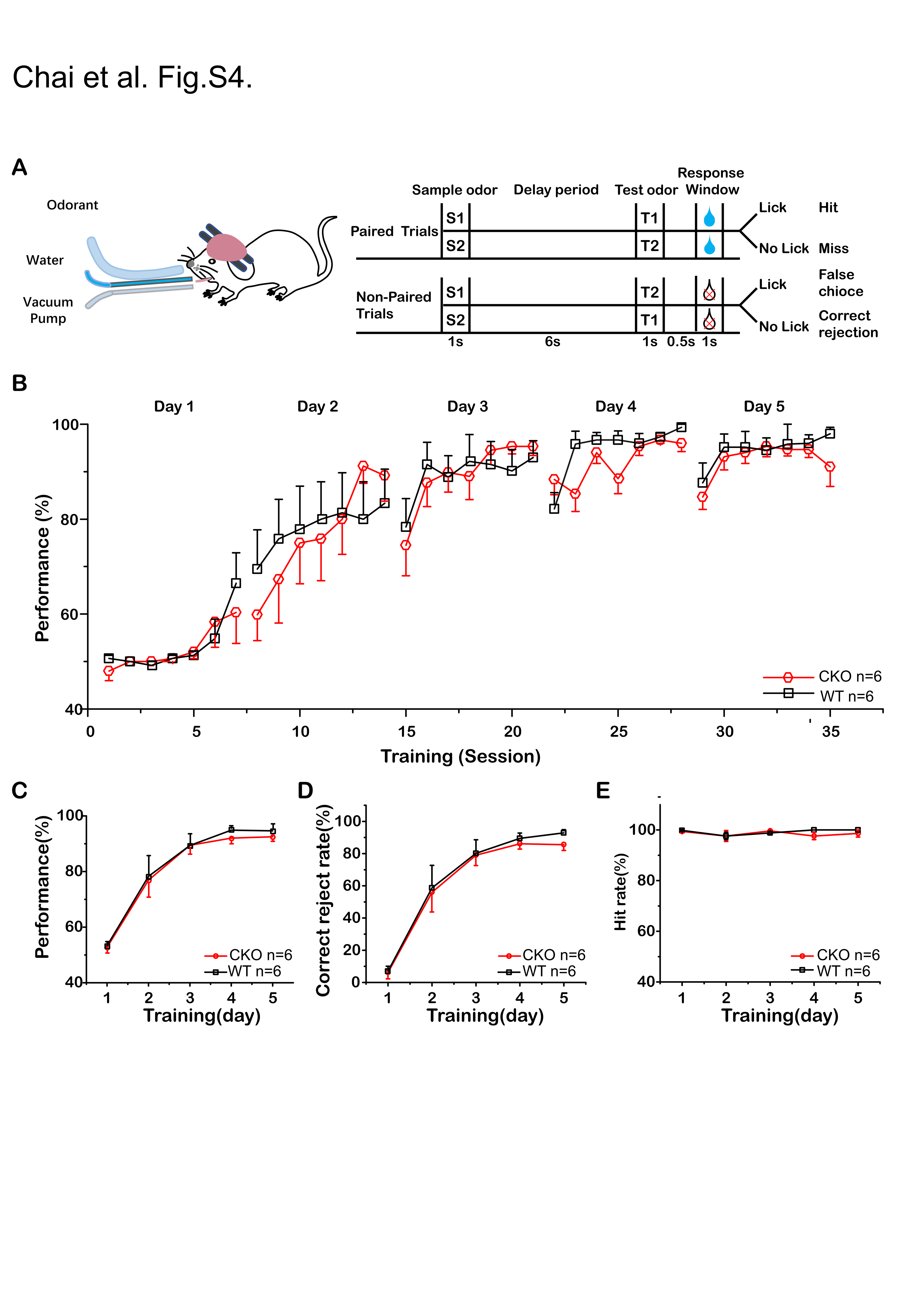


**Supplementary Figure 4. An odor GO/NO GO task for testing working memory was intact in CA1 PTEN conditional knockout (CKO) mice.** (A) Behavioural paradigm of odor GO/NO GO task. (B) An odor GO/NO GO task for testing working memory was examined during consecutive 5-days of training. The correct performance (%) in the GO task was not significant different between CA1 PTEN CKO and WT mice (n = 6/group, *P* > 0.05). (C) The averaged correct performance (%) over the training trials per day was also not significant different between PTEN CKO and WT mice (n = 6/group, *P* > 0.05). (D) The averaged correct reject rate (%) in the NO GO task was also not significant different between PTEN CKO and WT mice (n = 6/group, *P* > 0.05). (E) Hit rate (%) for the cue-induced responses were also not significant different between PTEN CKO and WT mice (n = 6/group, *P* > 0.05). Data presented as mean ± SEM. Statistical analysis was performed by using two-way ANOVA followed by Tukey’s post hoc analysis.
