# Supplementary material for: A Temporal Activity of CA1 Neurons Underlying Short-Term Memory for Social Recognition Altered in PTEN Mouse Models of Autism Spectrum Disorder
Source: Front Cell Neurosci. 2021 Jul 15;15:699315. doi: 10.3389/fncel.2021.699315 (PMC8319669; doi:10.3389/fncel.2021.699315)
Supplement: Supplementary file 5 [file Table_5.DOCX]

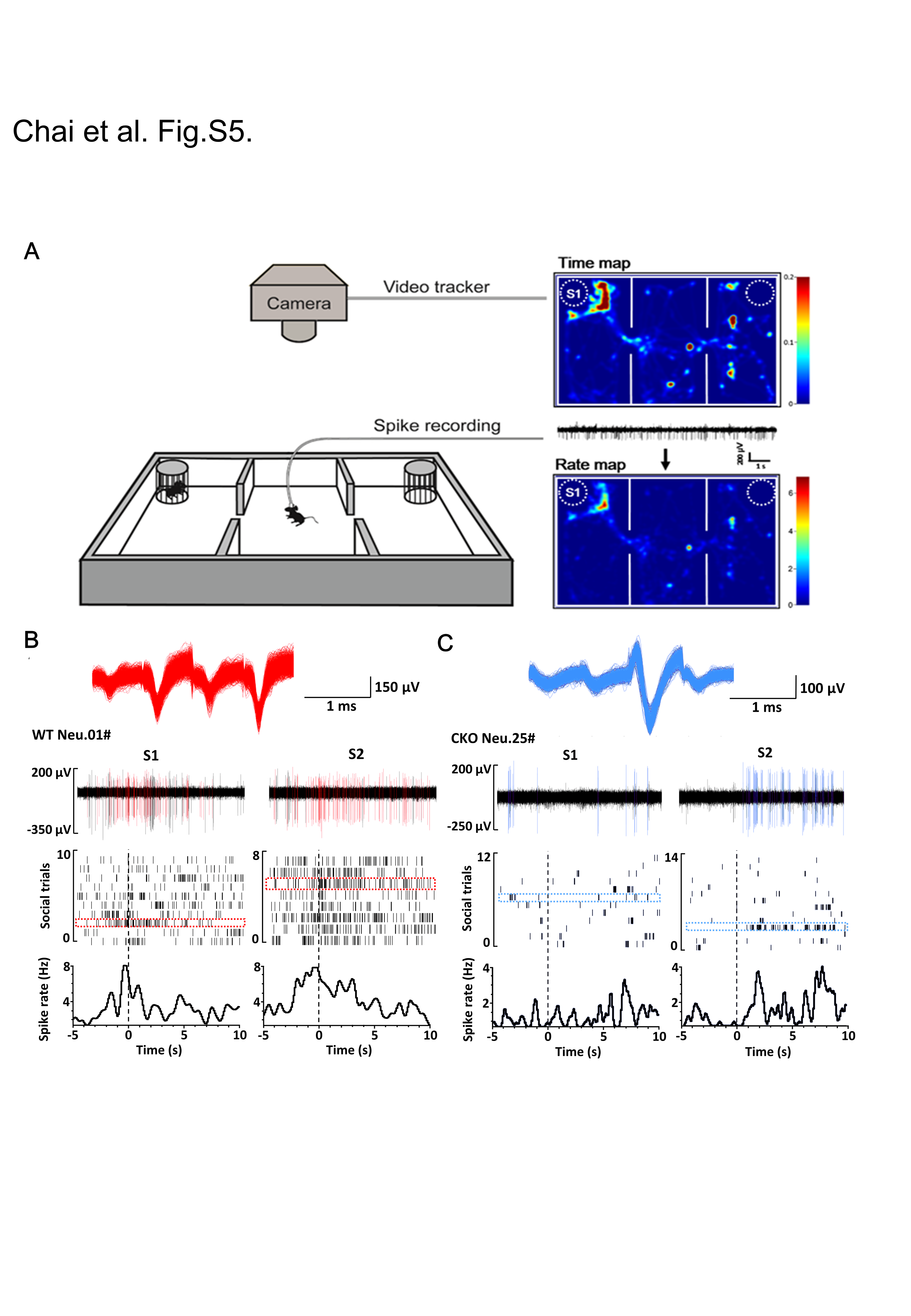


**Supplementary Figure 5. Multi-single unit recording of CA1 neuronal activity during social interaction test in PTEN CKO vs. WT mice.** (A) Left panels, we recorded the multi-single units in CA1 region and video-taped the behavioral trajectory from PTEN CKO and WT mice during the three-chamber’s and three-trials social interaction test. Right panels, a representative time map showing that a subject mouse explored the three-chambers and spent more time with S1 as indicated by the warmer colors. A representative rate map showing that a subject mouse explored the three-chamber and CA1 neurons fired at higher frequencies with S1 as indicated by the warmer colors. (B) Spike waveform (top), raster plots (middle) and peri-stimulus time histogram (PSTH, bottom) of continuous recording for a representative CA1 neuron aligned to the time of social exploration initiated by WT mouse with S1 (10 times) and S2 (8 times) respectively in trial 3. Red rectangles in raster plots highlight corresponding spike waveforms. Note the neuron showed peak response during social recognition. (C) Spike waveform (top), raster plots (middle) and PSTH (bottom) of continuous recording for a representative CA1 neuron aligned to the time of social exploration initiated by CKO mouse with S1 (12 times) and S2 (14 times) respectively in trial 3. Blue rectangles in raster plots highlight corresponding spike waveforms.
