# Supplementary material for: A Temporal Activity of CA1 Neurons Underlying Short-Term Memory for Social Recognition Altered in PTEN Mouse Models of Autism Spectrum Disorder
Source: Front Cell Neurosci. 2021 Jul 15;15:699315. doi: 10.3389/fncel.2021.699315 (PMC8319669; doi:10.3389/fncel.2021.699315)
Supplement: Supplementary file 6 [file Table_6.DOCX]

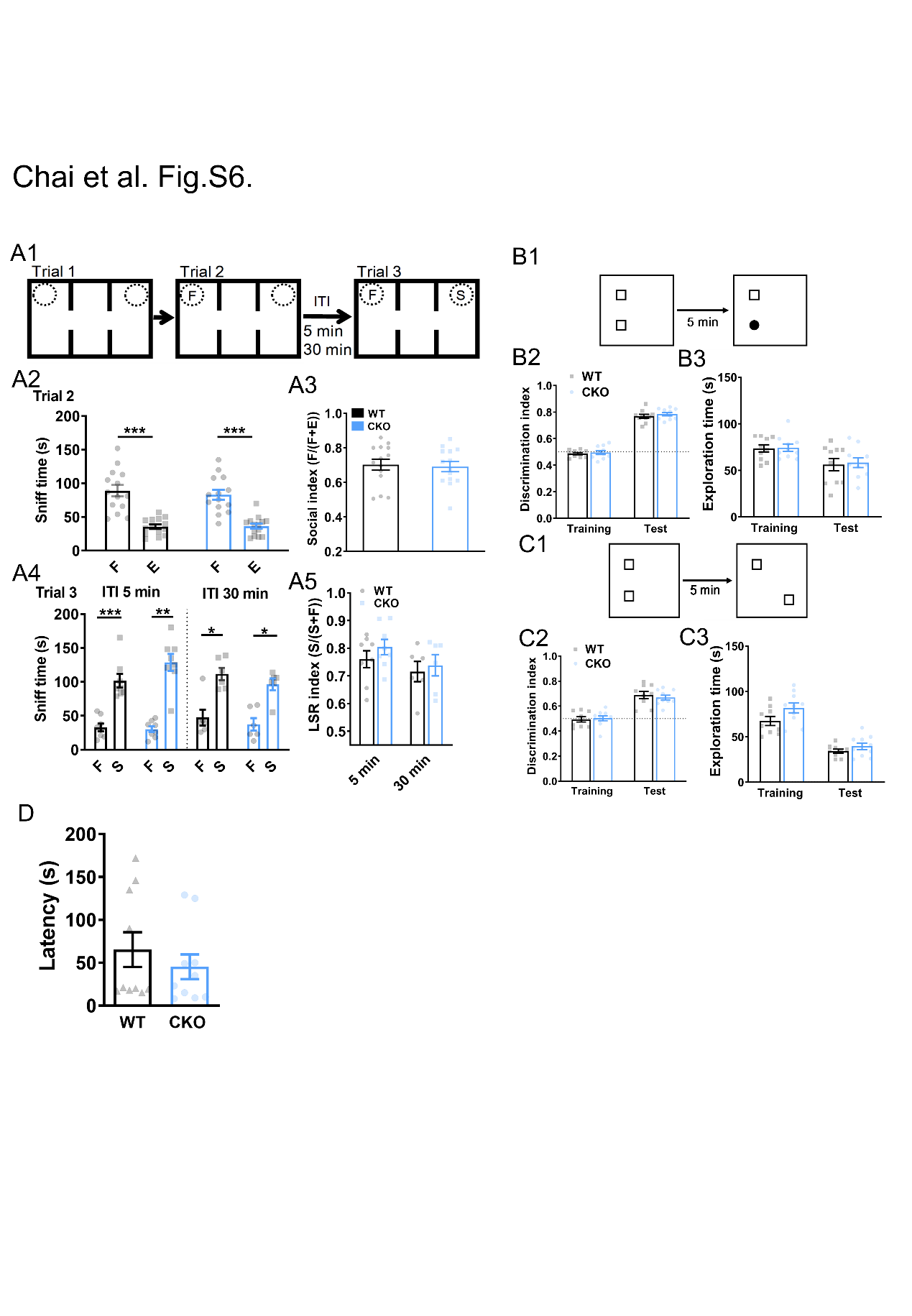


**Supplementary Figure 6. Social recognition, object recognition and olfaction test in conditional forebrain PTEN knockout (CKO) mice.** (A1) Experimental paradigm (A2) PTEN CKO and WT mice explored significantly more with familiar littermate (F) in trial 2 (n = 14 for WT and CKO; WT, t = 5.626, ****P* < 0.0001; CKO, t = 5.821, ****P* < 0.0001). (A3) Social index was similar between WT and CKO (n = 14 for WT and CKO; t = 0.2696, *P* = 0.7895). (A4) PTEN CKO and WT mice explored significantly more with stranger (S) in trial 3, regardless of ITI for 5 or 30 min (ITI 5 min, n = 8 for both groups; Wilcoxon test, WT, *P* = 0.0078; CKO, t = 7.4205, *P* = 0.000013; ITI 30 min, n = 6 for both groups; Wilcoxon test, WT, *P* = 0.0313; CKO, *P* = 0.0312) . (A5) Social recognition in trial 3 by exploring F *vs.* S were not significantly different between PTEN CKO and WT mice, regardless of the ITI for 5 or 30 min (ITI 5 min, n = 8 for both groups; t = 1.074, *P* = 0.5112; ITI 30 min, n = 6 for both groups; t = 0.4315, *P* = 0.6752; ***P* < 0.01; ****P* < 0.001). (B1) Experimental paradigm for shape-dependent object recognition. (B2) PTEN CKO mice exhibited normal shape-dependent object recognition as compared with WT mice (n = 10/group; Test, t = 0.8445, *P* = 0.4095). (B3) Total exploration time was similar between WT and CKO (n = 10/group; Wilcoxon test, Test, *P* = 0.9102). (C1) Experimental paradigm for position-dependent object recognition. (C2) PTEN CKO mice showed normal position-dependent object recognition relative to WT mice (n = 10/group; Test, *P* = 0.5485). (C3) Total exploration time was similar between WT and CKO (n = 10/group; Wilcoxon test, Test, t = 1.225, *P* = 0.2371). (D) There was no significant difference in olfaction test between PTEN CKO and WT mice (n = 10/group, t = 1.259, *P* = 0.2218; *P* > 0.05). Data presented as mean ± SEM. Statistical analysis was performed by using Mann Whitney’s U test, Wilcoxon test and student’s *t* test.
