# Supplementary material for: A Temporal Activity of CA1 Neurons Underlying Short-Term Memory for Social Recognition Altered in PTEN Mouse Models of Autism Spectrum Disorder
Source: Front Cell Neurosci. 2021 Jul 15;15:699315. doi: 10.3389/fncel.2021.699315 (PMC8319669; doi:10.3389/fncel.2021.699315)
Supplement: Supplementary file 7 [file Table_7.DOCX]

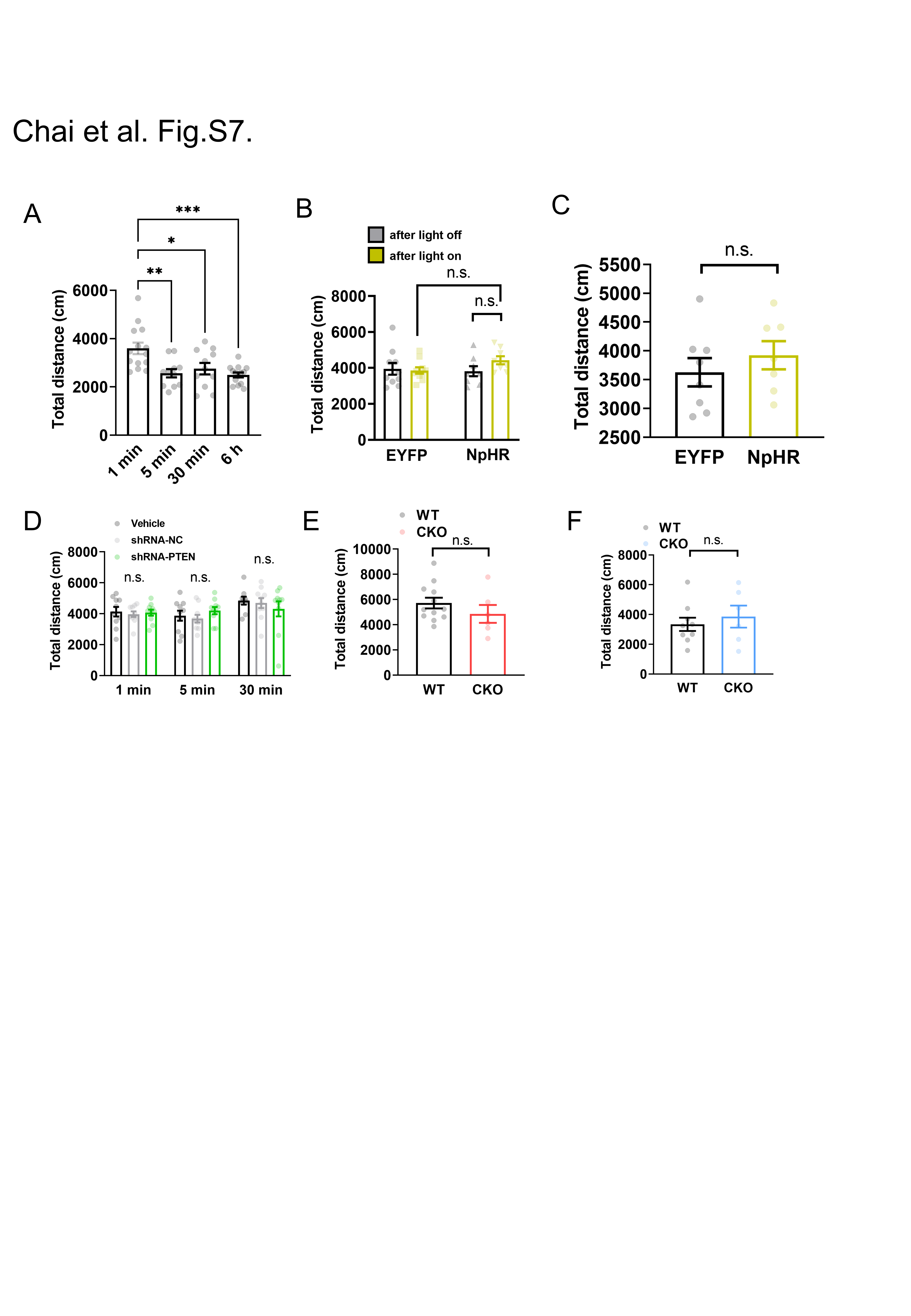


**Supplementary Figure 7. Total distance traveled during trial 3 for short-term memory guided social recognition.** (A) The total distance traveled during trial 3 in naïve mice was significantly higher in ITI 1 min group compared to that in ITI 5 min, ITI 30 min and ITI 6 h groups. But it was not significantly different in ITI 5 min group compared to that in ITI 30 min and ITI 6 h groups (n = 14 for 1 min; n = 11 for 5 min; n = 11 for 30 min; n = 14 for 6 h; *F*_(3,46)_ = 2.635, *P* = 0.0004. Tukey's post hoc test. 1 min vs. 5 min, *P* = 0.0030; 1 min vs. 30 min, *P* = 0.0201; 1 min vs. 6 h, *P* = 0.0006; 5 min vs. 30 min, *P* = 0.9163; 5 min vs. 6 h, *P* = 0.9940; 30 min vs. 6 h, *P* = 0.7836; **P* < 0.05; ***P* < 0.01; ****P* < 0.001). (B) The total distance traveled during trial 3 was not significantly different between EYFP and NpHR groups with light on during ITI 5 min. It was also not significantly different between NpHR group with light off and light on (n = 10 for EYFP, n = 8 for NpHR; EYFP light on vs. NpHR light on, t = 1.997, *P* = 0.0631; NpHR light off vs. NpHR light on, t = 1.623, *P =* 0.1486). (C) The total distance traveled during trial 3 was not significantly different between EYFP and NpHR groups with light on during trial 2 (n = 8 for EYFP, n = 7 for NpHR; t = 0.8502, *P* = 0.4106). (D) The total distance traveled during trial 3 was not significantly different among Vehicle (0.01 M PBS), shRNA-NC and shRNA-PTEN groups with ITI 1 min (n = 10/group; *F*_(2,27)_ = 2.257, *P* = 0.8895. Tukey's post hoc test. Vehicle vs. shRNA-NC, *P* = 0.8812; Vehicle vs. shRNA-PTEN, *P* = 0.9823; shRNA-NC vs. shRNA-PTEN, *P* = 0.9517.), ITI 5 min (n = 10/group; *F*_(2,27)_ = 0.6648, *P* = 0.4058. Tukey's post hoc test. Vehicle vs. shRNA-NC, *P* = 0.8671; Vehicle vs. shRNA-PTEN, *P* = 0.6805; shRNA-NC vs. shRNA-PTEN, *P* = 0.3795), and ITI 30 min (n = 10/group, but n = 9 in Vehicle; Kruskal-Wallis test, *P* = 0.8766. χ2 = 0.2634. Dunn's multiple comparisons test, 30min Vehicle vs. 30 min shRNA-NC, *P* > 0.9999; 30min Vehicle vs. 30min shRNA-PTEN，*P* > 0.9999; 30min shRNA-NC vs. 30min shRNA-PTEN, *P* >0.9999). (E) The total distance traveled during trial 3 was not significantly different between WT and CA1 PTEN CKO groups (n = 6 for WT, n = 12 for CKO; t = 1.102, *P* = 0.2866). (F) The total distance traveled during trial 3 was not significantly different between WT and PTEN CKO groups (n = 9 for WT, n = 7 for CKO; t = 0.6404, *P* = 0.5330). Data presented as mean ± SEM. Statistical analysis was performed by using Kruskal-Wallis test followed by Dunn’s post hoc analysis, student’s *t* test or one-way ANOVA followed by Tukey’s post hoc analysis.
